# Double-branch feature fusion transformer for hyperspectral image classification

**DOI:** 10.1038/s41598-023-27472-z

**Published:** 2023-01-06

**Authors:** Lanxue Dang, Libo Weng, Yane Hou, Xianyu Zuo, Yang Liu

**Affiliations:** 1 Henan Key Laboratory of Big Data Analysis and Processing, Henan University, Kaifeng, 475001 China; 2 Henan Province Engineering Research Center of Spatial Information Processing, Henan University, Kaifeng, 475001 China; 3 School of Computer and Information Engineering, Henan University, Kaifeng, 475001 China

**Keywords:** Computational science, Computer science, Optical spectroscopy

## Abstract

Deep learning methods, particularly Convolutional Neural Network (CNN), have been widely used in hyperspectral image (HSI) classification. CNN can achieve outstanding performance in the field of HSI classification due to its advantages of fully extracting local contextual features of HSI. However, CNN is not good at learning the long-distance dependency relation and dealing with the sequence properties of HSI. Thus, it is difficult to continuously improve the performance of CNN-based models because they cannot take full advantage of the rich and continuous spectral information of HSI. This paper proposes a new Double-Branch Feature Fusion Transformer model for HSI classification. We introduce Transformer into the process of HSI on account of HSI with sequence characteristics. The two branches of the model extract the global spectral features and global spatial features of HSI respectively, and fuse both spectral and spatial features through a feature fusion layer. Furthermore, we design two attention modules to adaptively adjust the importance of spectral bands and pixels for classification in HSI. Experiments and comparisons are carried out on four public datasets, and the results demonstrate that our model outperforms any compared CNN-Based models in terms of accuracy.

## Introduction

Due to the advancement of current imaging spectrometry techniques, hyperspectral image (HSI) contains rich spectral and spatial information with high spectral and spatial resolution^1^, so pixel-level classification can be achieved^2,3^. HSI are widely used in many fields, such as atmospheric environment research^4^, precision agriculture^5–7^, and ocean research^8^. However, there is a lot of redundant information in the spectral bands of HSI and the difficulty in obtaining samples of HSI^9^ brings difficulties to the classification of HSI. In early studies of HSI classification, some machine learning-based approaches, such as SVM^10^, k-NN^11^, and multilayer perceptron (MLP)^12^, were used for HSI classification. However, most of them focus on the spectral information of HSI without taking full advantage of the spatial information of HSI. Although some methods based on morphological profiles^13^ and Gabor feature^14^ are presented to extract spatial features, the classification accuracy is still unsatisfactory. This is because these methods can only extract low-level features and the limited training samples of HSI.

The rapid development of deep learning techniques has brought the more diversified effective approaches for HSI classification. Deep learning follows an “end-to-end” design philosophy and can automatically extract linear and nonlinear features. Compared with traditional methods, which require a large amount of domain expert knowledge, deep learning methods can avoid designing manual features and improve the generalization ability of the model. Some deep learning-based models, such as Stacked Autoencoder (SAE)^15^, Recurrent Neural Network (RNN)^16,17^, and deep belief network (DBN)^18^, have been merged and successfully applied to HSI classification. Hang et al.^17^ proposed a model consisting of two RNN layers that can extract complementary information from non-adjacent spectral bands of HSI. RNN-based models can extract spectral features by considering the spectral dimension of HSI as a sequence, but they are prone to gradient vanishing, and difficult to learn long-distance dependency relations^19^.

Convolutional Neural Network (CNN) can effectively extract the spatial features of HSI, due to its powerful ability to extract local contextual information. A lot of CNN-based models have appeared in recent years. Hu et al.^20^ firstly used CNN for HSI classification and proposed a 1DCNN-based model, which includes multiple 1DCNNs and only considers the spectral features of HSI. Although the performance of 1DCNN-based model is poor, it has promoted the development of CNN-based models in HSI classification. Subsequently, a series of CNN-based models taking account of spectral and spatial features of HSI has been developed. Zhong et al.^21^ presented a 3DCNN-based model through a 3D convolution kernel to extract spectral-spatial features of HSI. Paoletti et al.^22^ designed a 2DCNN-based model based on deep pyramid network^23^, which can improve the classification performance by stacking a large number of convolution kernels. Li et al.^24^ proposed a 3DCNN-based Double-Branch model, where the two branches extract spectral and spatial features of HSI respectively. Gao et al.^25^ proposed a small convolution and feature reuse (SC-FR) module by combining cascaded 1 $\times$ 1 convolutional layers and cross-layer connections. There is only one 3 $\times$ 3 convolution in the model to extract spatial features of hyperspectral images. Dang et al.^26^ proposed a dual-path and small-convolution-based module (DPSC) for the extraction of spatial and spectral features from hyperspectral images. Both of these models are based on small convolutions to build lightweight models. Chang et al.^27^ proposed a method based on a consolidated convolutional neural network (C-CNN) composed of 2DCNN and 3DCNN to learn the spatial-spectral features and abstract spatial features of hyperspectral images. Shi et al.^28^ proposed a model based on multi-scale feature fusion and double attention mechanism to extract features from hyperspectral images. Although the CNN-based models have made some progress in HSI classification, the performance of them is still insufficient. First, HSI usually contains hundreds of bands and the spectral characteristics of some ground objects are extremely similar. CNN is not good at learning long-distance dependency relations of spectral bands^29^, and cannot accurately classify such objects. Secondly, the size of the convolution kernel in the CNN-Based model is usually small, and it is easy to extract the local features rather than the global features of the entire neighborhood pixel blocks. These problems cause the bottleneck of the CNN-based model in the classification of HSI. Improving the performance of CNN-based model in HSI classification becomes very important and meaningful.

The development of Transformer^30^ techniques brings a new idea to HSI classification, which was originally used in the field of Nature Language Processing (NLP). Transformer is very effective at processing sequence data^30^, which can extract global features of input data through a self-attention mechanism, and can better learn long-distance dependency relations of input data^31,32^. Dosovitskiy et al.^32^ proposed the first Transformer-based model for computer vision, Vision Transformer(ViT), and achieved good results. This model extracts global features by segmenting the image into patches. We can apply Transformer to extract features of HSI by regarding HSI as a sequence. HSI can be regarded as sequences in two ways. One is that the spectral bands of HSI are rich and continuous, so the entire spectral bands can be treated as a sequence. The other is that the spectral vector of each pixel can be considered as a word vector in the NLP field^31^, because of each pixel representing a ground object. However, simply applying the Transformer model, for example, vision transformer (ViT)^32^, into HSI classification still has many problems. First of all, segmenting the neighborhood pixel blocks with a fixed size like ViT makes it difficult to extract the low-level features of the input data^33^. Next, segmenting neighborhood patches only in the spatial dimension still fails to learn long-range dependency relations for the spectral features of HSI.In view of this, this paper proposes a Double-Branch Feature Fusion Transformer (denotedas DBFFT) model for HSI classification. The proposed model adopts two branches to extract spectral and spatial features of HSI respectively. The spectral branch consists of a spectral attention module and Transformer encoder block. The spatial branch is made up of a spatial attention module and Transformer encoder block. In addition, a feature fusion layer is designed between these two branches to fuse spectral and spatial features. The outputs obtained by the two branches are fused by addition operation, and finally used for classification. The main contributions of this paper can be described as follows:

- The proposed model extracts the spectral features and spatial features of HSI respectively through a Double-Branch structure. In the two branches, according to the sequence characteristics of hyperspectral images, Pixel-wise embedding and Band-wise embedding are adopted to effectively extract the long-distance dependency relations of spectral dimension of HSI and the global spatial feature of HSI.
- We design a CNN-based spectral attention module and a spatial attention module, which can adaptively adjust the importance of spectral and spatial features of the input data, and extract rich spectral and spatial features.
- Our proposed model adopts label smooth techniques to alleviate the overfitting phenomenon of the model when the number of samples is small. In addition, we design a feature fusion layer to fuse the features extracted by the two branches to improve the performance of the model.

The remainder of this paper is organized as follows. In Sect. “Methodology”, we describe the details of our proposed model. In Sect. “Experiments results and analysis”, we present and analyze the experimental results, in addition to analyzing the factors that affect the performance of the model. In Sect. “Conclusion”, we give conclusions and present directions for future work.

## Methodology

### Overview of the proposed model

We set the HSI to be a data cube with length S, width M, and number of bands C. We take each labeled pixel as the center and segment a 3D cube of size $H\times H\times C$ called the neighborhood pixel block, where H is the length and width of the neighborhood pixel block, C represents the number of spectral bands of the HSI. We take neighborhood pixel blocks as input to the model to fully utilize the spectral and spatial information of HSI.

**Figure 1:**
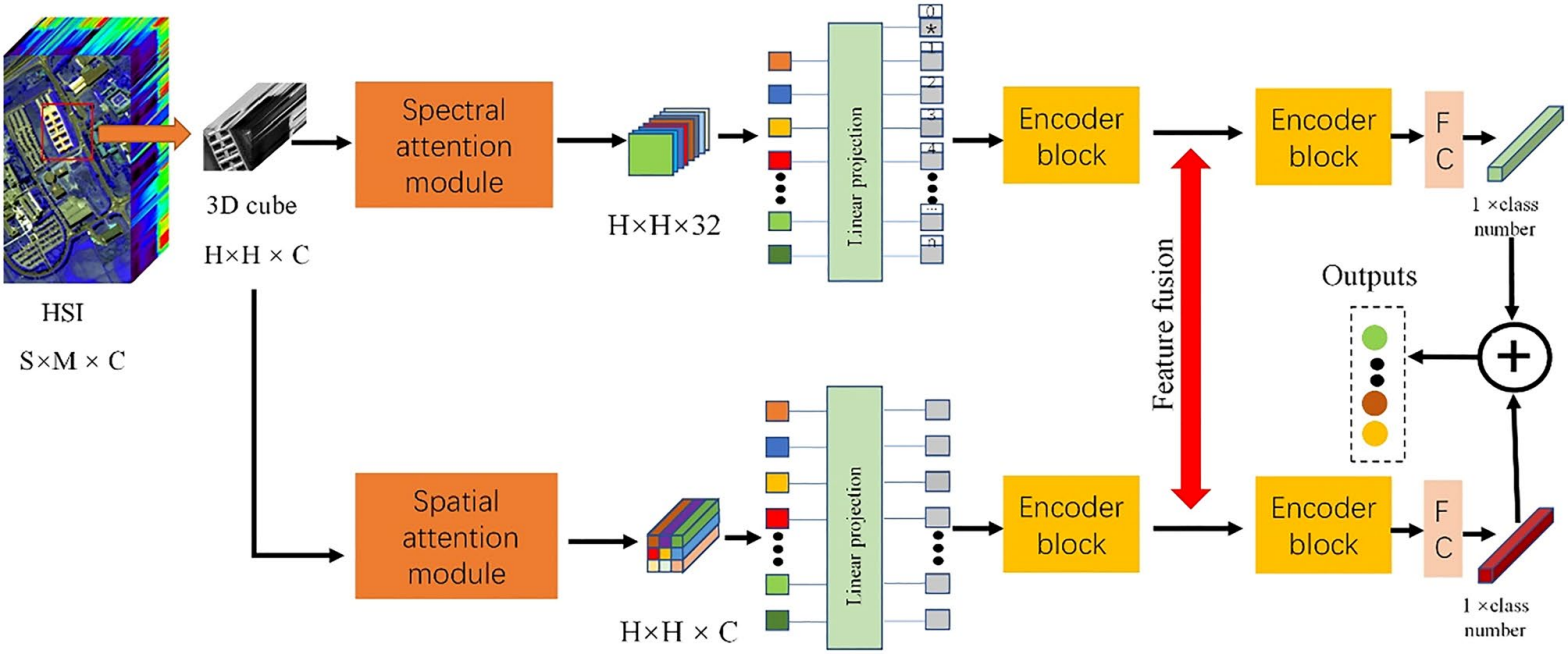
The structure of the DBFFT. This model consists of two branches. The upper branch consists of a spectral attention module and Transformer encoder block to extract spectral features of HSI. The lower branch consists of spatial attention module and Transformer encoder block to extract spatial features of HSI.

Figure 1 shows the overall structure of our proposed model. The model contains two branches to extract spectral features and spatial features of HSI respectively. We take the upper branch as the spectral branch and the lower branch as the spatial branch. The spectral branch consists of the spectral attention module and the Transformer encoder block. The spatial branch is made up of a spatial attention module and a Transformer encoder block. Inspired by CrossViT^34^, we add a feature fusion layer between the two branches to fuse the spatial features and the spectral features.

The spectral branch first uses the spectral attention module to extract the rich spectral features of the neighborhood pixel blocks of size $H\times H\times C$. Then the dimension of the spectral dimension is reduced from $C$ to $k$ to remove redundant information, and a new feature map of size $H\times H\times k$ will be gotten. We set $k$ = 32. After that, the feature map is segmented according to the spectral dimension to obtain $k$ patches of size $H\times H$, which are flattened and processed by linear projection to generate a sequence of shape (batch size, $k+1$, $M$), where M represents the length of the vector in the sequence. This sequence will be used as input to the Transformer encoder block of the spectral branch. The spectral branch of our proposed model can utilize self-attention to extract global features, capturing the long-distance dependency relations of the spectral dimension.

The spatial branch first uses the spatial attention module to extract the rich spatial features of the neighborhood pixel blocks of size $H\times H\times C$ to obtain a new feature map of size $H\times H\times C$. The feature map is segmented by pixel, and $H\times H$ vectors of length $C$ are obtained and processed by linear projection to generate a sequence of shape (batch size, $H\times H$, M). Use this sequence as the input to the Transformer encoder block. The spatial branch can extract the global spatial features of HSI.

Finally, the outputs of the two branches are fused to fuse spectral features and spatial features. We will describe the abovementioned parts in detail in the following sections.

### Depth-wise separable convolution

**Figure 2:**
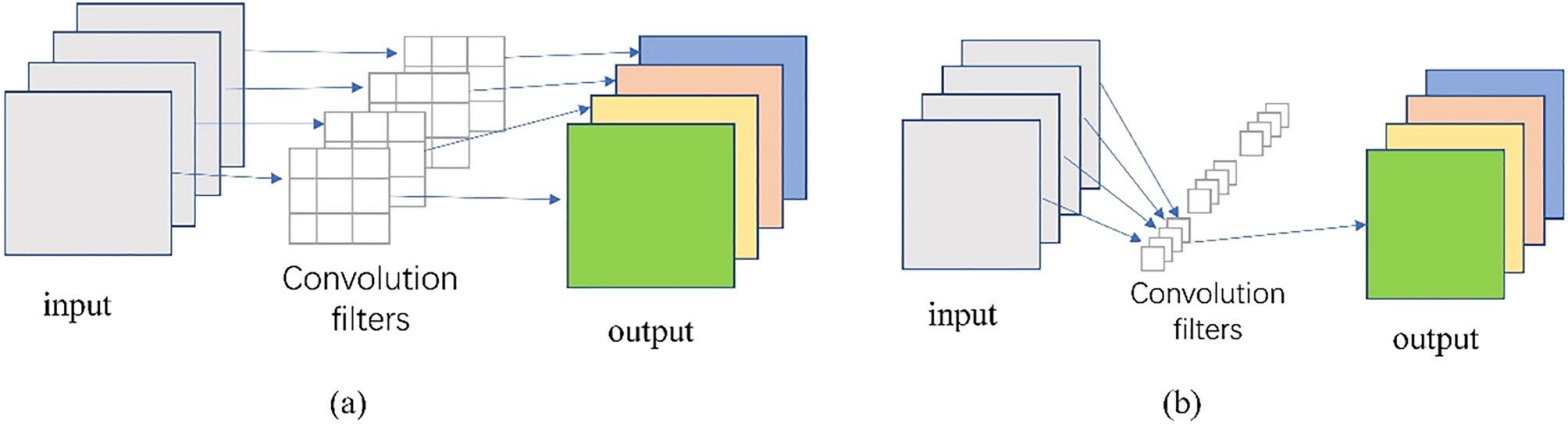
The depth-wise separable convolution consists of two parts: (**a**) depth-wise convolution. (**b**) 1 × 1 convolution.

As shown in Fig. 2, the depth-wise separable convolution consists of a depth-wise convolution layer and a 1 × 1 convolution layer. Depth-wise separable convolution can extract rich low-level features from HSI at the beginning of the entire attention module. Each convolution kernel in the depth-wise convolution only extracts spatial features in one spectral dimension. The 1 × 1 convolution fuses the features of different spectral bands to obtain a feature map. Since the spectral information of HSI is rich and redundant, the use of depth-wise separable convolution can reduce the redundant information of the extracted spectral dimension and the interference of redundant bands on feature extraction.

### Spectral attention module

**Figure 3:**
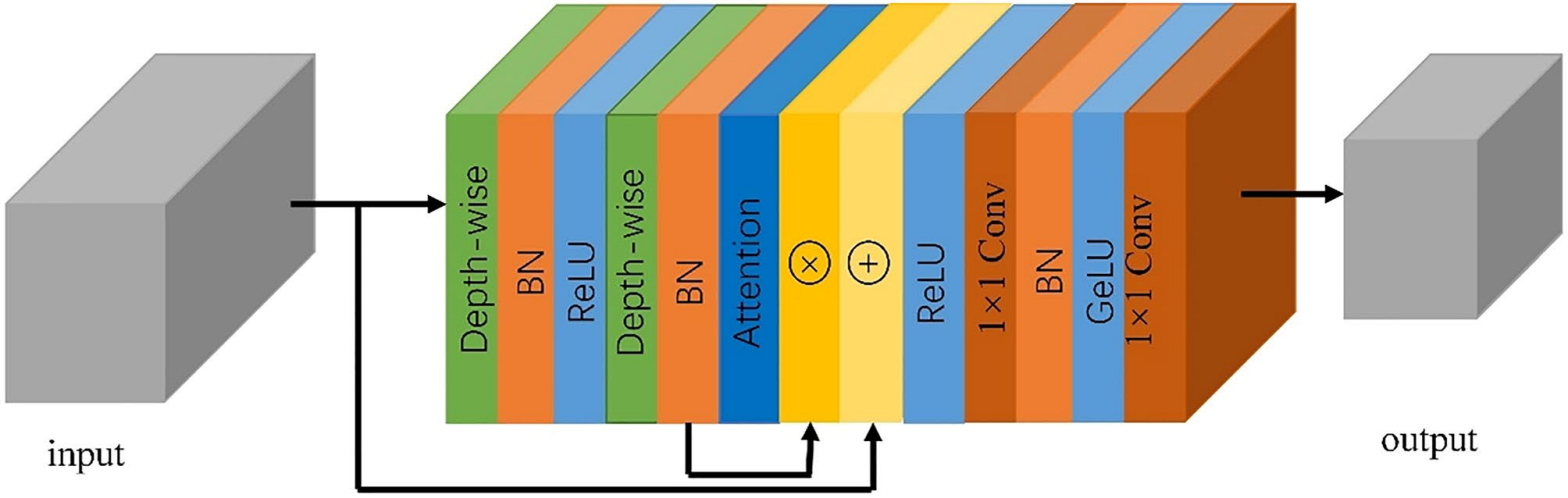
The structure of the attention module. The input of this model is the neighborhood pixel patch of the original hyperspectral image, and the output is the feature map.

The redundant spectral information of raw HSI data will interfere with the recognition of the model. Therefore, by processing the HSI with the spectral attention module, the influence of noise information on the model is reduced, and the redundant information of HSI is reduced. The framework of the module is shown in Fig. 3. We extract the pixel-centered neighborhood pixel block of shape $H\times H\times C$ as input, where $H$ represents the size of the neighborhood pixel block and $C$ represents the spectral dimension of the HSI. First, the low-level features of the neighborhood pixel blocks are extracted through two layers of depth-wise separable convolution layers. Second, the spectral attention ${\varvec{s}}{\varvec{e}}\in {{\varvec{R}}}^{1\times 1\times {\varvec{C}}}$ is generated by spectral attention to adjust the importance of each spectral band, and then the obtained feature map is fused with the original data to retain the original spectral and spatial features. Finally, the spectral features of the spectral dimension are fused through two 1 × 1 convolution layers with GeLU. The above process does not change the size of the neighborhood pixel blocks, but it can reduce the spectral dimension and redundant spectral features.

**Figure 4:**
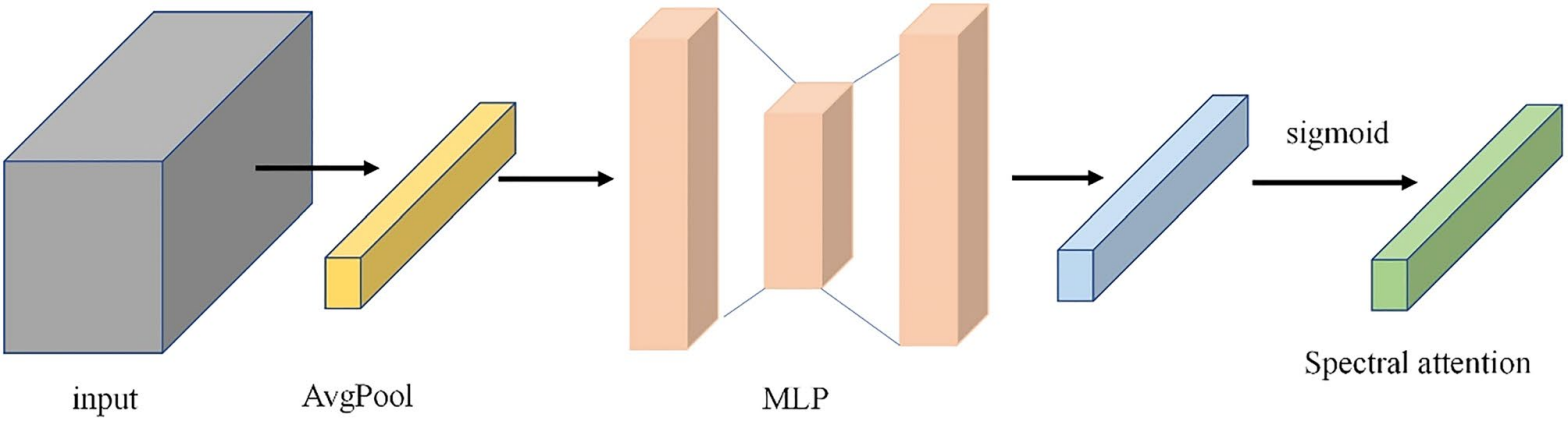
Generate spectral attention. This module contains a global average pooling and a multilayer perceptron (MLP) consisting of two fully connected layers.

The spectral attention mechanism can automatically adjust the importance of different spectral bands for classification and reduce the interference of useless bands to the model. Figure 4 shows the whole process of generating spectral attention. Inspired by SE-block^35^, our computational process for generating spectral attention ${\varvec{s}}{\varvec{e}}$ is defined as follows:

1 $$h_{\left( k \right)}^{avg} = \frac{1}{H \times H}\mathop \sum \limits_{i = 1}^{H} \mathop \sum \limits_{j = 1}^{H} E\left( {k,i,j} \right)$$

2 $$\varvec{se} = \sigma _{2} \left( {FC_{2} \left( {\sigma _{1} \left( {FC_{1} \left( {h^{{avg}} } \right)} \right)} \right)} \right)$$

where $E$ represents the obtained feature map after the neighborhood pixel block is processed by two depth-wise separable convolution layers, $E\left(k,i,j\right)$ represents the value of the position (i, j) of the k-th channel of the feature map E, ${h}^{avg}$ represents the result of global average pooling, ${h}_{\left(k\right)}^{avg}$ represents the value of the kth channel of ${h}^{avg}$, and ${\sigma }_{1}$ and ${\sigma }_{2}$ represent ReLU and sigmoid activation functions, respectively. ${FC}_{1}$ and ${FC}_{2}$ are two fully connected layers. The first layer reduces the dimension from M to M/r, and the second layer increases the dimension from M/r to M. We set r to be 16.

After spectral attention ${\varvec{s}}{\varvec{e}}$ and feature map ${F}_{1}$ are multiplied by band, the importance of different bands can be automatically adjusted.

### Spatial attention module

Since we use the neighborhood pixel block as the input of the model, we usually regard the labels of all pixels of the neighborhood pixel block as the label of the center pixel. It will lead to the interference of the information of the pixels with different labels of the original center pixel to the model^36^. Therefore, we use a spatial attention module to enhance the information of pixels that are helpful for classification and weaken the information of pixels that interfere with classification. The framework of the spatial attention module is the same as Fig. 3, the difference lies in the part that generates the attention, which will generate a spatial attention. And this module does not change the spectral dimension of the input data.

**Figure 5:**
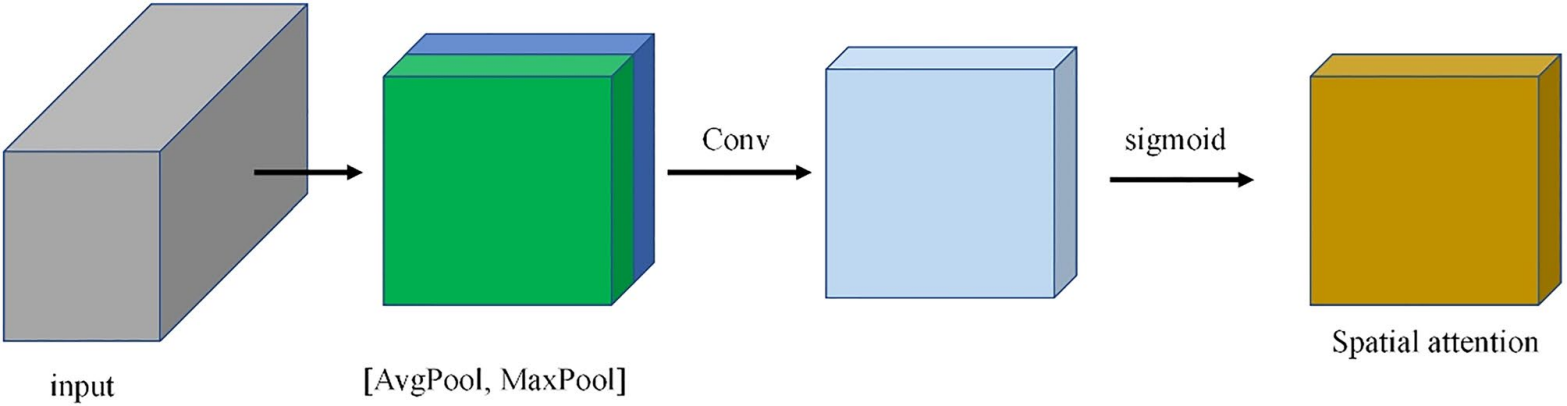
Generate spatial attention. This module concatenates the outputs of global average pooling and global max pooling through a convolutional layer.

Figure 5 shows the whole process of generating spatial attention. Inspired by CBAM^37^, we first perform global average pooling and global max pooling in the spectral dimension to generate ${s}^{avg}$ and ${s}^{max}$ of shape $H\times H\times 1$. The calculation process of this part is described in Eqs. (3) and (4).

3 $$S_{{\left( {{\text{i}},j} \right)}}^{avg} = \frac{1}{c}\mathop \sum \limits_{k = 1}^{c} F\left( {\kappa ,{\text{i}},j} \right)$$

4 $$s^{max} = Max\left( {\text{F}} \right)$$

where $F$ represents the feature map obtained after the neighborhood pixel block is processed by two depth-wise separable convolution layers in the spatial branch, F(κ,i,j) represents the value of the position (i,j) of the feature map F on the kth channel, ${s}^{avg}$ represents the result of global average pooling, ${S}_{\left(i,j\right)}^{avg}$ represents the value of the position (i, j) of ${s}^{avg}$, $Max\left(\mathrm{F}\right)$ represents the maximum value of all channels of each pixel in the feature map F..

Then, we concatenate ${s}^{avg}$ and ${s}^{max}$. After processing through a convolutional layer and a sigmoid activation function, the spatial attention ${\varvec{s}}{\varvec{a}}\in {{\varvec{R}}}^{{\varvec{H}}\times {\varvec{H}}\times 1}$ is obtained.

5 $$sa^{^{\prime}} = {\text{conv}}\left( {\left[ {s^{avg} ,{ }s^{max} } \right]} \right){ }$$

6 $$sa = {\text{sigmoid}}\left( {sa^{^{\prime}} } \right)$$

After the spatial attention ${\varvec{s}}{\varvec{a}}$ and the feature map ${F}_{2}$ are multiplied by pixels, the importance of different pixels for classification can be automatically adjusted.

### Pixel-wise embedding and Band-wise embedding

The classic ViT structure segments the image into patches according to a fixed size. When ViT has simply been applied to segment the image, it is not suitable for the characteristics of HSI because each pixel on the HSI represents a ground object. Meanwhile, such a segmentation method cannot learn the long-distance dependency relations of the spectral bands of HSI. To better combine the characteristics of HSI, we adopt Pixel-wise embedding and Band-wise embedding in the two branches to better learn the global features of HSI. In the spatial branch, we perform Pixel-wise embedding on the feature maps of the spatial attention module. We segment the feature map of shape $H\times H\times C$ by pixel to generate $H\times H$ vectors of length $C$. Finally, the length of the vector is adjusted to M by the full connection layer processing, and M is set to 64. We did not add position embedding to the vectors because the CNN can encode the absolute position of the image^38^.

**Figure 6:**
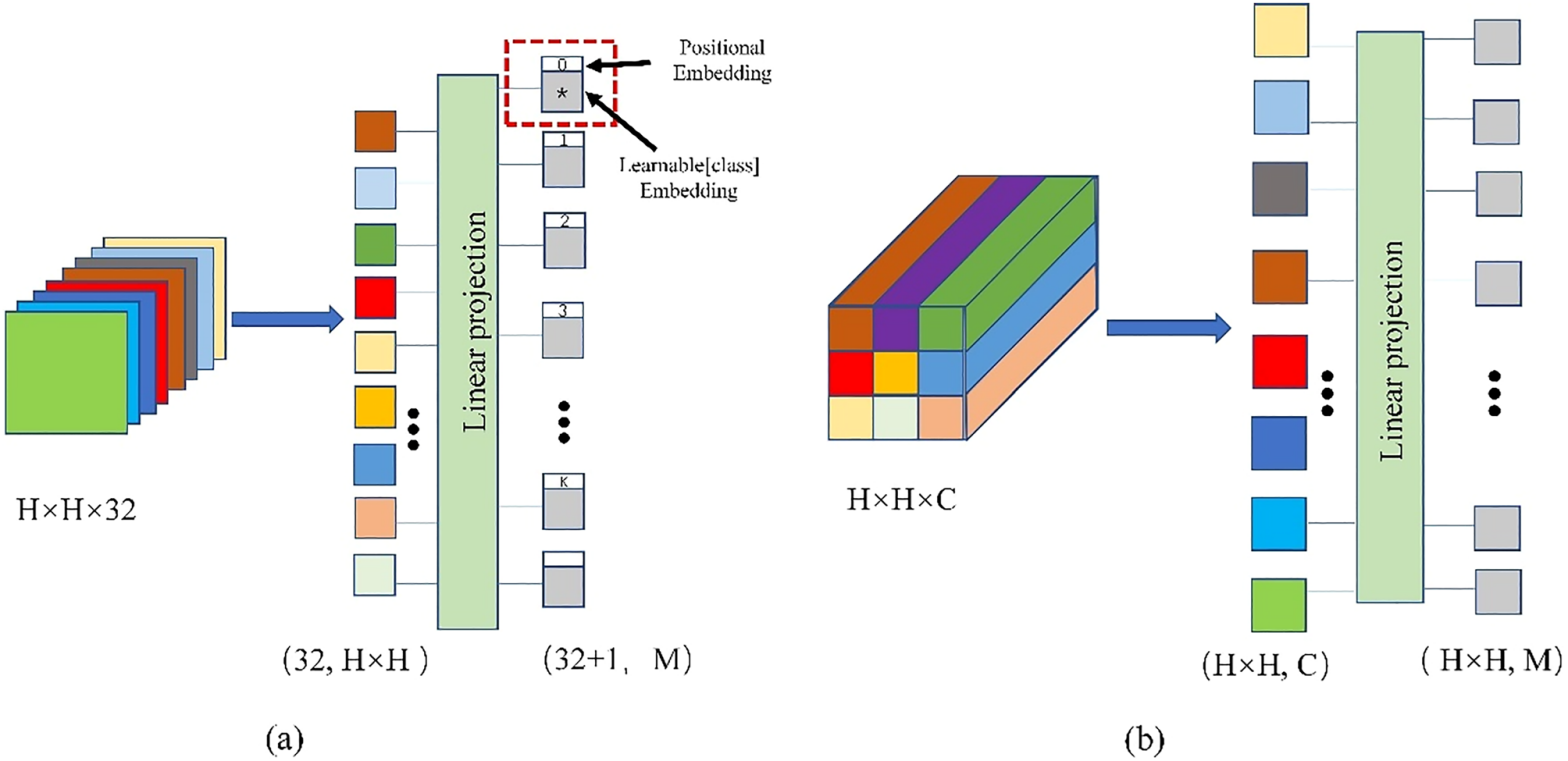
Two ways of linear projection methods. (**a**) Band-wise embedding (**b**) Pixel-wise embedding.

Considering that the spectral dimension information of the feature map is rich and continuous, we use Band-wise embedding to segment the HSI according to the spectral dimension, and then flatten the two-dimensional patch of each band. After that, the vector of output length M is processed through the fully connected layer as the input of the Transformer. This can learn long-distance dependency relations in the spectral dimension of HSI. Lastly, the generated sequence is used as the input of the transformer, after adding the positional embedding and the learnable embedding. Figure 6 illustrates how Pixel-wise embedding and Band-wise embedding process feature maps into sequences. Although the linear projection methods of the two branches are different for the characteristics of HSI, the length of the vector after linear projection is the same, which is to facilitate the fusion of features at the feature fusion layer.

### Transformer encoder block

**Figure 7:**
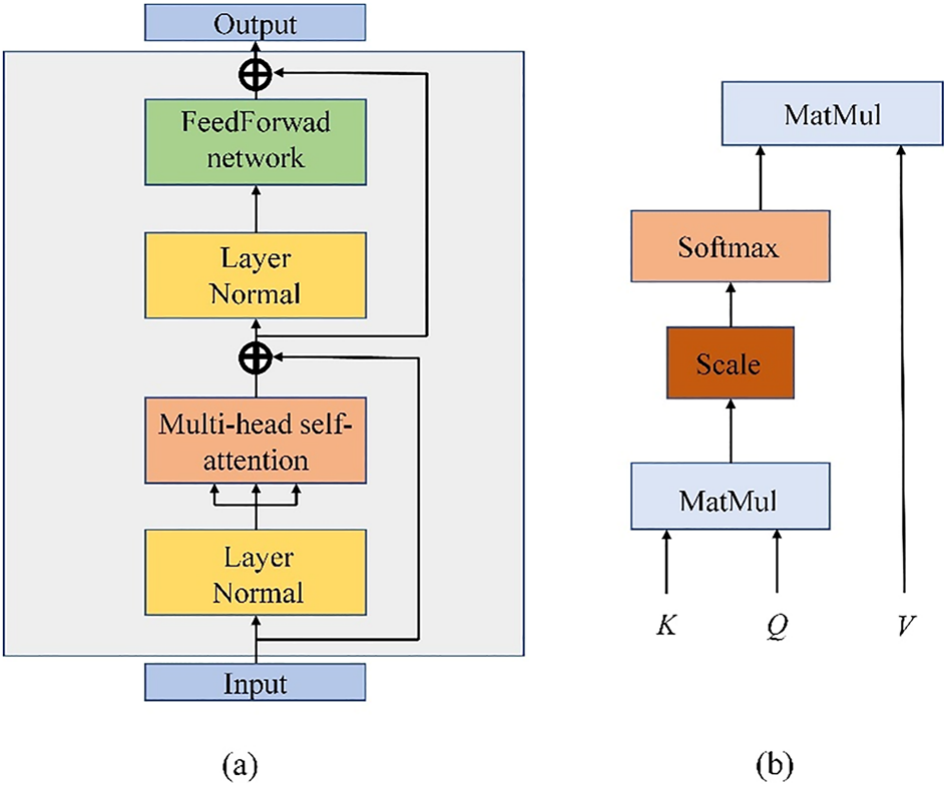
Structure of the Transformer encoder block and the illustration of the self-attention mechanism. (**a**) Transformer encoder block. (**b**) self-attention mechanism.

Each branch of our proposed model contains two Transformer encoder blocks respectively to extract global features of HSI. As shown in the Fig. 7a, each transformer encoder block consists of a multi-head self-attention mechanism sublayer and a Feedforward network sublayer, and each sublayer has LayerNormalization and residual connections. Figure 7b shows the processing of the self-attention mechanism in Transformer. The self-attention mechanism can extract the global features of the input sequence, and its calculation process is described in Eq. (7).

7 $${\text{z }} = {\text{ Attention}}\left( {{\text{Q}},{\text{K}},{\text{V}}} \right){\text{softmax}}\left( {\frac{{Qk^{T} }}{{\sqrt {{\text{d}}_{k} } }}} \right)V$$

where $K$, $Q$, $V$ are obtained by multiplying the input sequence with ${w}^{Q}$, ${w}^{K}$ and ${w}^{V}$ respectively. ${d}_{k}$ represents the dimension of the vector in K, whose role is to obtain a stable gradient by scaling^19^. Multi-head self-attention mechanism is to concatenate the outputs obtained by multiple self-attentions. Multiple heads are computed independently and each head has a different focus on the sequence. The formula is defined as follows:

8 $${\text{Mulit}} - {\text{Head attention}}\left( {K,Q,V} \right) \, = {\text{concat}}({\text{z}}_{1} ,{\text{z}}_{2} , \ldots ,{\text{z}}_{h} )W^{o}$$

where ${W}^{o}$ is a matrix and $h$ represents the number of heads.

The Feedforward network consists of two fully connected layers and a GeLU activation function, which can further transform the features learned in self-attention mechanism. Equation (9) gives its calculation process.

9 $${\text{Feedforward network}}\left( {input} \right) = {\text{FC}}\left( {\sigma \left( {{\text{FC}}\left( {input} \right)} \right)} \right)$$

where $\sigma$ denotes GeLU activation function.

### Feature fusion layer

Our proposed model extracts spatial and spectral features of HSI on two branches separately. Inspired by CrossViT^34^, we add a feature fusion layer between the two branches to fuse the features extracted by the two branches. Specifically, we consider exchanging the class tokens (i.e. the Learnable Embedding illustrated in Fig. 6) of the output sequence of the Transformer encoder block of the spectral branch and the first vector of the output sequence of the Transformer encoder block of the spatial branch. It is because the Transformer-based model uses the first vector of the output sequence to classify. Thus, we can think of this vector as a summary of the entire sequence^34^. Therefore, the class token of the output sequence of the spectral branch contains rich spectral features, and the first vector of the output sequence of the spatial branch contains rich spatial features. By exchanging these two vectors, the fusion of spectral and spatial features can be facilitated.

### Label smooth

When the training samples that are used to train the model are insufficient, the generalization ability of the model will be reduced, which will lead to overfitting of the model. In practical applications, this problem of insufficient HSI samples is also very common. In order to decrease the influence of the overfitting phenomenon on the model, we introduce a regularization technique label smooth to alleviate it.

First, we change each label to use a one-shot representation. The vector ${y}_{n}$ represents the one-shot representation of each label y, its dimension is S dimension, where S represents the number of classes, and the value on the vector is 1 when n = y, otherwise it is 0. Then, we add noise $\varepsilon$ to the label as follows:

10 $$y_{n}^{^{\prime}} = \left( {1 - \varepsilon } \right)y_{n} + \frac{\varepsilon }{S}$$

where ${y}_{n}^{^{\prime}}$ is the new label obtained after label smooth, $\varepsilon$ is the noise.

The model tends to become more "confident" during the training process, but the lack of training set samples and mislabeling of the dataset will cause the model to generate more misclassifications in the test set. By adding noise to each label, the model becomes "unconfident", the generalization ability of the model is improved, and the overfitting of the model is alleviated.

## Experiments results and analysis

### Data sets description

We adopt four public datasets: Kennedy Space Center (KSC), Salinas (SA), University of Pavia (PU), and Houston 2013(HU) to evaluate the performance of the proposed model.

*Kennedy Space Center (KSC)*: This dataset was collected by AVIRIS sensors over the Kennedy Space Center (KSC) in Florida, USA. This dataset contains 512 × 614 pixels, and after removing the noise-affected bands, a total of 176 bands are available for experiments. It has a spatial resolution of 18 m and a wavelength range of 400 to 2500 nm. It contains a total of 13 land cover categories with a total of 5211 labeled pixels. The training samples, validation samples and test samples for each category are shown in the Table 1.

**Table 1:** Number of training, validation, and test samples for KSC dataset.

NO	Class	Train	Val	Test
1	Scrub	39	38	684
2	Willow swamp	12	13	218
3	CP hammock	13	13	230
4	Slash pine	13	13	226
5	Oak/broadleaf	8	9	144
6	Hardwood	12	11	206
7	Swamp	6	5	94
8	Graminoid marsh	22	22	387
9	Spartina marsh	26	26	468
10	Cattail marsh	20	21	363
11	Salt marsh	21	21	377
12	Mud flats	26	25	452
13	Water	46	47	834
	Total	264	264	4683

*Salinas (SA)*: This dataset was collected by AVIRIS sensors over the Salinas Valley in California. This dataset contains 512 × 217 pixels, and after removing the noise-affected bands, a total of 204 bands are available for experiments. It has a spatial resolution of 3.7 m and a wavelength range of 400 to 2500 nm. It contains a total of 16 land cover categories with a total of 54,129 labeled pixels. The training samples, validation samples and test samples for each category are shown in the Table 2.

**Table 2:** Number of training, validation, and test samples for SA dataset.

NO	Class	Train	Val	Test
1	Brocoli_green_weeds_1	21	20	1968
2	Brocoli_green_weeds_2	37	38	3651
3	Fallow	20	20	1936
4	Fallow_rough_plow	14	14	1366
5	Fallow_smooth	27	27	2624
6	Stubble	40	40	3879
7	Celery	36	36	3507
8	Grapes_untrained	113	113	11,045
9	Soil_vinyard_develop	62	63	6078
10	Corn_senesced_green_weeds	33	33	3212
11	Lettuce_romaine_4wk	11	11	1046
12	Lettuce_romaine_5wk	20	19	1888
13	Lettuce_romaine_6wk	10	9	897
14	Lettuce_romaine_7wk	11	11	1048
15	Vinyard_untrained	73	73	7122
16	Vinyard_vertical_trellis	18	19	1770
	Total	546	546	53,037

*University of Pavia (PU)*: This dataset was collected by ROSIS sensors over the University of Pavia in northern Italy. This dataset contains 610 $\times$ 340 pixels, and after removing noise-affected bands, a total of 103 bands are available for experiments. It has a spatial resolution of 1.3 m and a wavelength range of 430 to 860 nm. It contains a total of 9 land cover categories with a total of 42,776 labeled pixels. The training samples, validation samples and test samples for each category are shown in the Table 3.

**Table 3:** Number of training, validation, and test samples for PU dataset.

NO	Class	Train	Val	Test
1	Asphalt	67	66	6498
2	Meadows	186	187	18,276
3	Gravel	21	21	2057
4	Trees	31	31	3002
5	Sheets	13	14	1318
6	Bare soils	51	50	4928
7	Bitumen	14	13	1303
8	Bricks	37	37	3608
9	Shadows	9	10	928
	Total	429	429	41,918

*Houston 2013 (HU)*: This dataset was collected by the ITRES CASI-1500 sensor over the University of Houston campus, which is provided by the 2013 IEEE GRSS Data Fusion Competition ^39^. This dataset contains 349 × 1905 pixels. This dataset has 144 spectral bands for experiments. It contains a total of 15 land cover categories with a total of 15,029 labeled pixels. The training samples, validation samples, and test samples for each category are shown in Table 4.

**Table 4:** Number of training, validation, and test samples for the HU dataset.

NO	Class	Train	Val	Test
1	Healthy grass	13	13	1225
2	Stressed grass	13	13	1228
3	Synthetic grass	7	7	683
4	Trees	13	12	1219
5	Soil	12	13	1217
6	Water	3	4	318
7	Residential	13	13	1242
8	Commercial	13	12	1219
9	Road	13	13	1226
10	Highway	13	12	1202
11	Railway	12	13	1210
12	Parking lot1	13	12	1208
13	Parking lot2	5	5	459
14	Tennis court	4	5	419
15	Running Track	7	7	646
	Total	154	154	14,721

For deep learning methods, the more samples are used for training, the better the performance of the model will be gotten. It means that the training of the model will be more time-consuming as well as requiring more labeled pixels. Our proposed model can still maintain the optimal performance in the case of small samples. Therefore, for KSC, we consider 5% of the samples for training, 5% for validation, and the rest for testing. For PU, SA, and HU, we consider 1% of samples for training, 1% for validation, and the rest for testing.

### Experimental setup

The software environment for our experiments is Python version 3.7.0 and the deep learning framework in PyTorch version 1.2.0. The hardware environment for our experiments is RTX2060 GPU with 6 GB RAM and AMD CPU R7-4800 at 2.9 GHz with 16 GB RAM. We choose SGD optimizer^40^ to optimize the training parameters of the model, and the loss function chooses the cross-entropy loss function. The learning rate is set to 0.001, 0.001, 0.01, and 0.001 on KSC, SA, PU, and HU respectively. The epoch on four datasets is set to 200.

In order to quantitatively evaluate the classification performance of the model, we choose OA (overall accuracy), AA (average accuracy), and kappa coefficient (κ) as the evaluation indicators of the model.

### Parameters setting

We analyze some factors that affect the training and performance of the model, which are batch size, learning rate, number of head and input size. To be fair, each of our subsequent experiments was repeated ten times, and the metrics used were the average of 10 experiments. We chose 10 different random seeds for 10 experiments to exclude variability due to random factors in the experiments.

**Figure 8:**
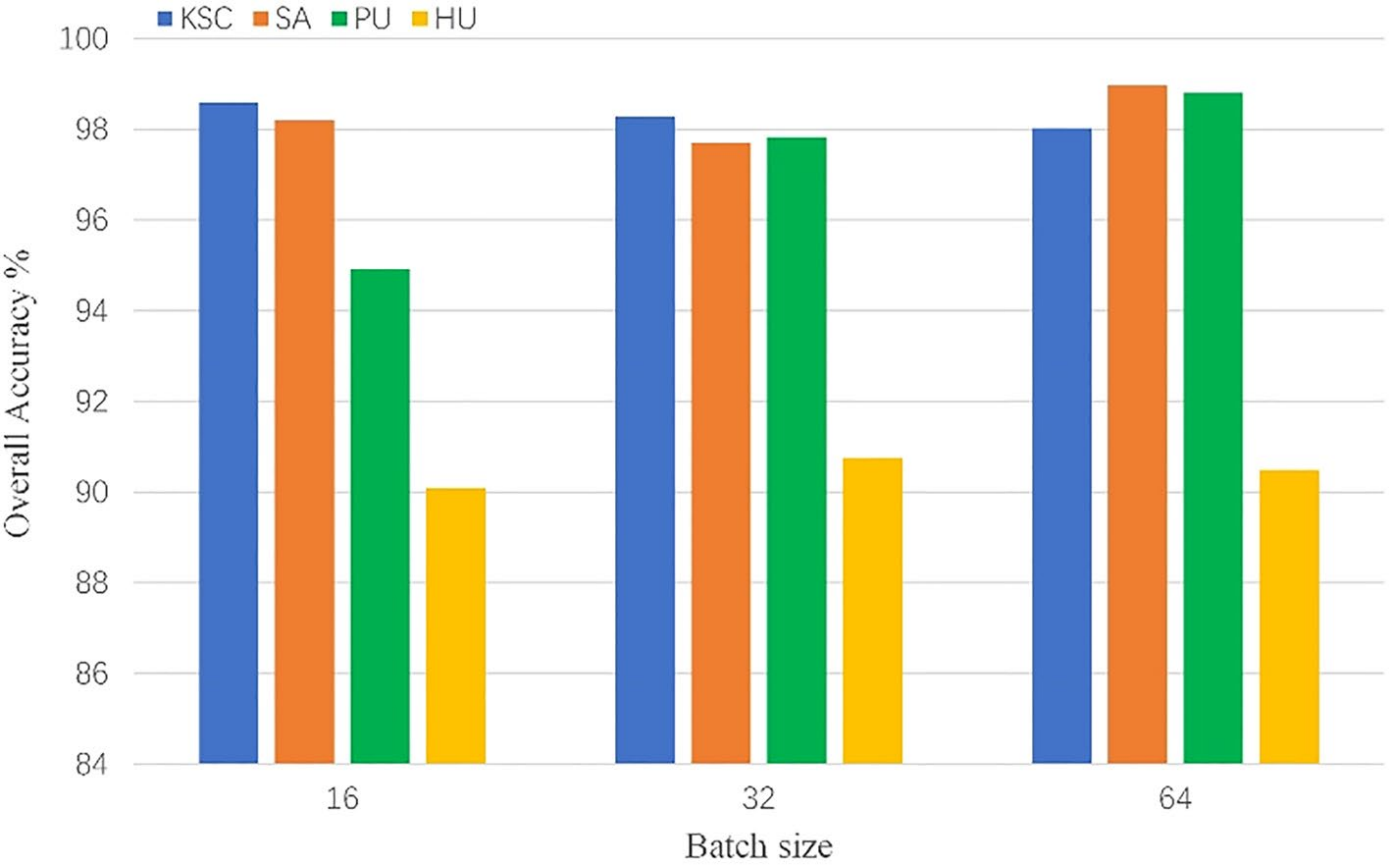
OA (%) of DBFFT with different batch size in the four datasets.

**Figure 9:**
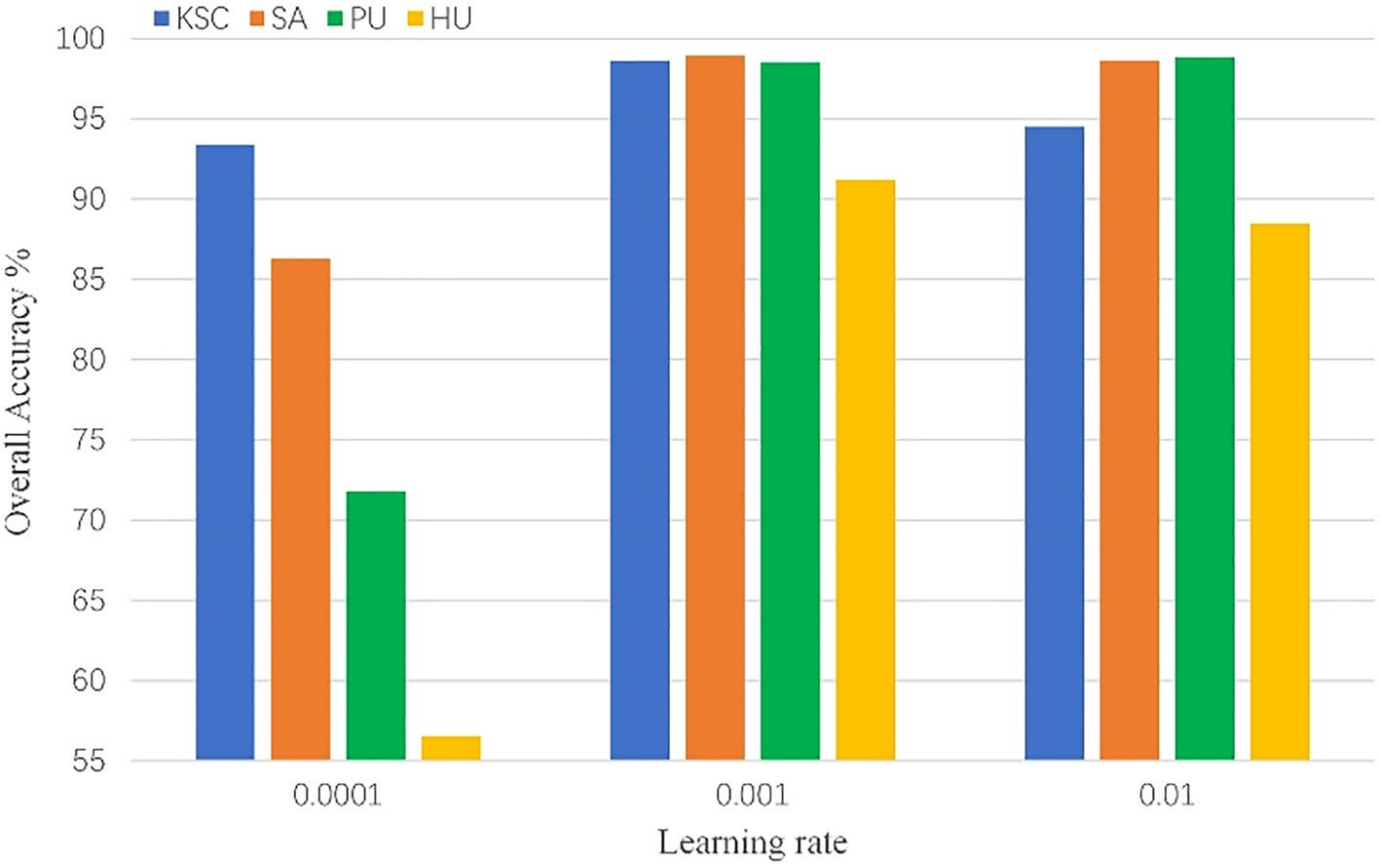
OA (%) of DBFFT with different learning rate in the four datasets.

**Figure 10:**
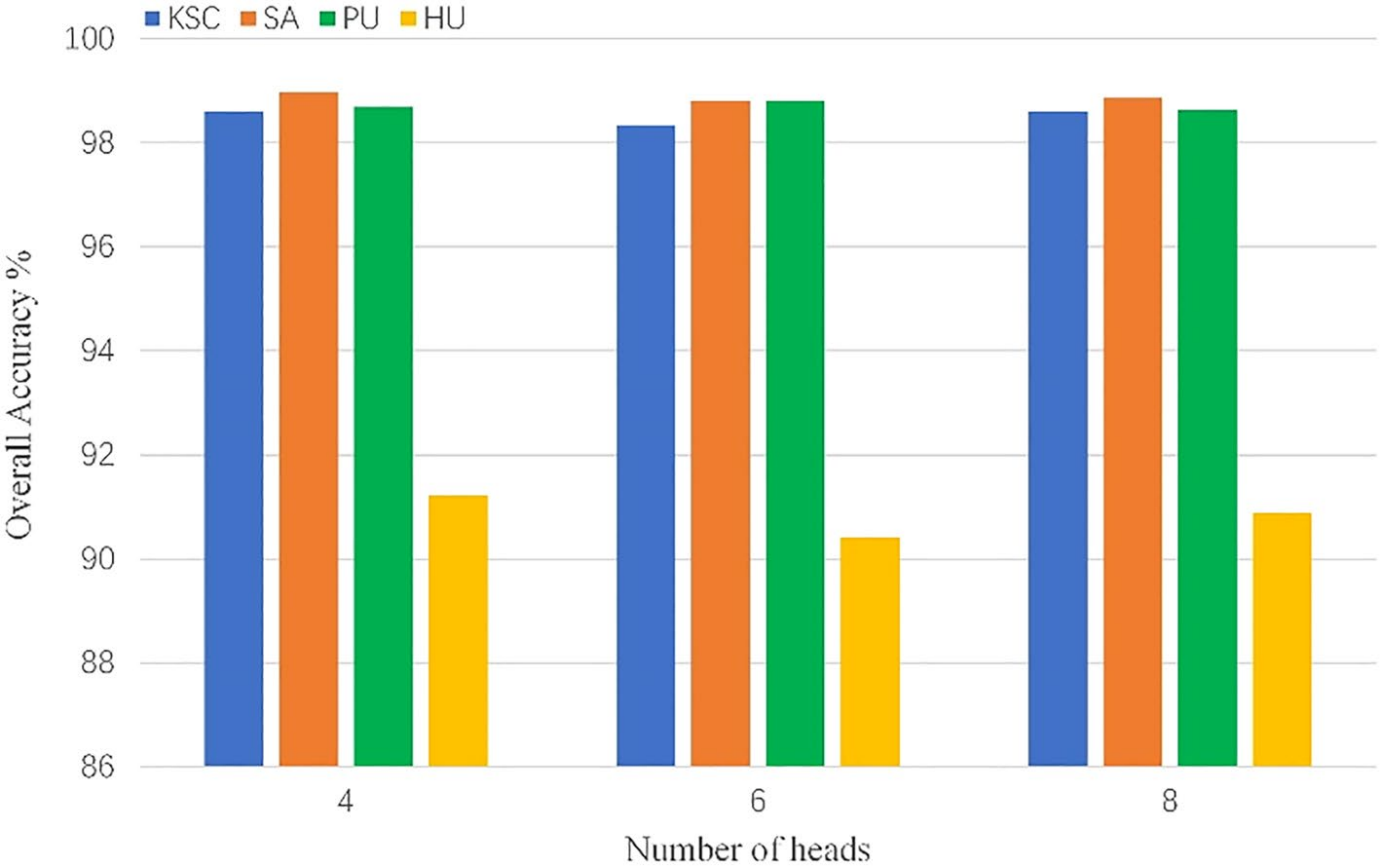
OA (%) of DBFFT with different number of heads in the four datasets.

**Figure 11:**
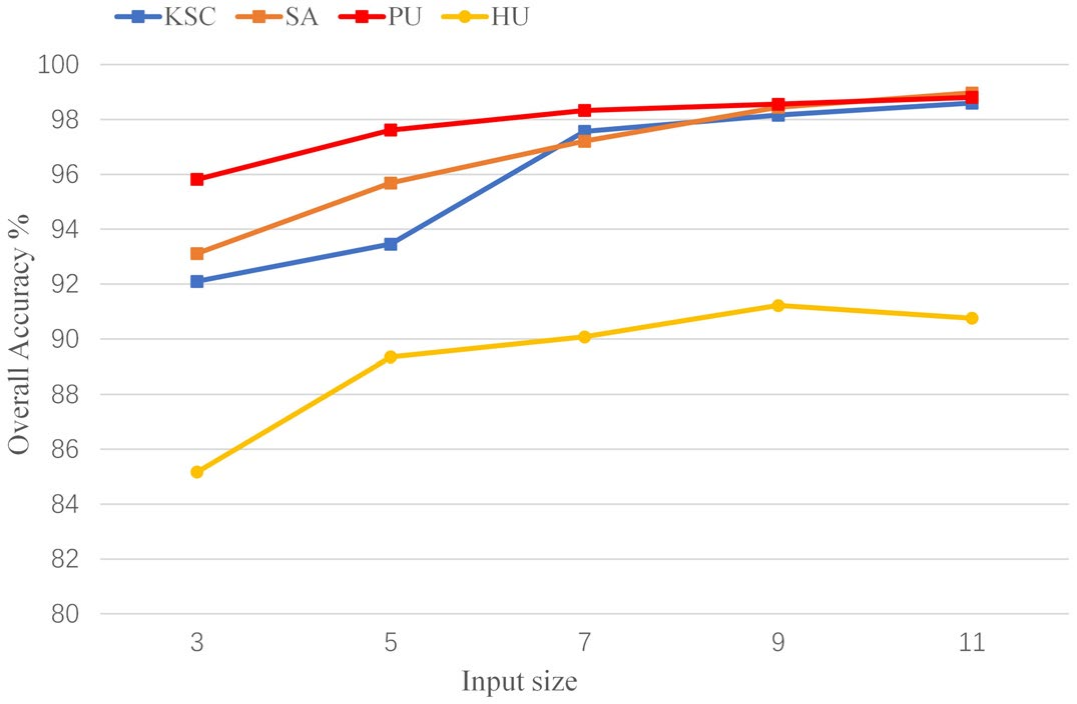
OA (%) of DBFFT with different input size in the four datasets.

1. *Batch size*: Batch size is important for model training, which affects the convergence of the model. We consider the sets of {16, 32, 64} for experiments. The results are shown in the Fig. 8, we can see that choosing the appropriate batch size for training is very important for the final performance of the model, so we chose to use 16 on KSC, 64 on SA, 64 on PU, and 32 on HU.
2. *Learning rate*: The learning rate affects the convergence speed of the model during training, and it plays an important role in the performance of the model. We choose a learning rate sets of {0.01, 0.001, 0.0001} for experiments. As shown in the Fig. 9, choosing different learning rates to train the model has a great impact on the final performance of the model. Based on the above results, we choose to use 0.001 on KSC, 0.001 on SA, 0.01 on PU, and 0.001 on HU, respectively.
3. *Number of heads*: Transformer's multi-head self-attention can extract the global relationship between vectors in the sequence. Different heads can extract different relationships between vectors and other vectors. We select a set of head numbers {4, 6, 8} to evaluate the effect of head count on the model. As shown in the Fig. 10, different head counts affect the performance of the model. We use 4 on KSC, 4 on SA, 6 on PU, and 4 on HU respectively, according to the experimental results.
4. *Input size*: The input size determines the spatial information that the model can use for classification. To better evaluate the effect of size on the model, we choose a set of sizes {3, 5, 7, 9, 11}. As shown in the Fig. 11, as the size increases, the OA of the model continues to increase.  In the HU dataset, the OA of size 11×11 is lower than the OA of size 9×9, but its value is still higher than that of sizes 3×3, 5×5, and 7×7. This indicates that the increase of spatial information can improve the information that can be mined by the model. We choose the size of $11\times 11$ as the input size of the model on the PU, KSC, SA datasets, and $9\times 9$ as the input size of the model on the HU dataset.

### Comparison results of different methods

In this section, our proposed model is compared with the traditional method MLP as well as five deep learning models, such as 1D-CNN^20^, M3D-DCNN^41^, pResNet^22^, SSRN^21^, DBDA^24^, SCFR^25^ and DPSCN^26^. Among these methods, except for MLP and 1D-CNN, the neighborhood pixel patch is used as the input of the model. The hyperparameters (such as input size, learning rate) and training skills (such as early stopping, learning rate dynamic adjustment) of all the models are set according to their original paper to ensure fairness. We repeat each group of experiments in the four datasets 10 times with randomly selected training samples to ensure the fairness of the experiments. Meanwhile, we will also report the mean and standard deviation for all the metrics. Now, we briefly introduce the methods mentioned above in the following.

1. *MLP*: It is a multilayer perceptron that consists of two fully connected layers and a ReLU.
2. *1D-CNN*: It consists of 1D convolutional layers and fully connected layers.
3. *M3D-DCNN*: This model extracts multi-scale information by combining multiple 3D convolution kernels of different sizes, and the size of the neighborhood pixel block is 7 × 7.
4. *pResNet*: This model is based on 2DCNN. By introducing a deep pyramid network^23^, the depth of the model is improved to extract rich spectral and spatial information. The size of the neighborhood pixel block is 11 × 11.
5. SSRN: This model consists of multiple spectral residual blocks and spatial residual blocks. The two residual blocks are based on ResNet and 3DCNN. The size of the neighborhood pixel block is 7 × 7.
6. *DBDA*: A 3DCNN-based Double-Branch model, each branch consists of DenseNet and attention mechanism. The size of the neighborhood pixel block is 9 × 9.
7. *SCFR*: This model is completely composed of 1 × 1 convolutions except that the first layer is composed of 3 × 3 convolution. The size of the neighborhood pixel block is 7 × 7.
8. *DPSCN*: This model is constructed by the dual-path small convolution (DPSC) module. DPSC module consists of 1 × 1 convolution and with a residual path and a density path. The size of the neighborhood pixel block is 9 × 9.

The classification results of different models on the four datasets are shown in Tables 5, 6, 7 and 8, and the best classification results are shown in bold. It can be seen that the performance of our proposed model is the best on all four datasets. MLP and 1D-CNN, which only utilize the spectral information of HSI, have the lowest performance on all four datasets. The accuracy of the model using spatial information is higher than the MLP and 1D-CNN, which proves the importance of spatial information for HSI classification. It is worth noting that the performance of M3D-DCNN is much lower than pResNet, SSRN, DBDA, and DBFFT on the Four datasets. The reason is that the depth of M3D-DCNN is shallow and it is difficult to extract deep features of HSI. Furthermore, in the case of small samples, M3D-DCNN overfits the training data. The pResNet model performs poorly on PU, KSC, and HU, and its OA on PU, KSC and HU is 4.23%, 2.76%,8.81% lower than DBFFT, respectively. The reason is that pResNet stacks a large number of convolution kernels, which leads to too many training parameters of the model, resulting in overfitting of the model in the case of a small sample. In addition, the over-reliance of the 2DCNN-based model on the spatial features of HSI also leads to the poor performance of the model. SCFR and DPSCN are mainly composed of 1 × 1 convolutions, and these two models utilize a small amount of 3 × 3 convolutions to extract spatial information. SCFR performed poorly on all four datasets, suggesting that SCFR did not extract enough spatial features. The performance of DPSCN on PU is close to DBFFT, and OA is only 0.08% lower than DBFFT, but on KSC, SA, and HU, OA is 2.28%, 4.9%, and 2.2% lower than DBFFT, respectively. This indicates the poor generalization ability of DPSCN. Both SSRN and DBDA are 3D-CNN based models, but their performance on all four datasets is much lower than that of our proposed model. DBDA, which is the same as our proposed model, is also a Double-Branch structure, but the OA on KSC, SA, PU, and HU is 1.35%, 1.01%, 0.16%, 0.9% lower than DBFFT, respectively. This illustrates the importance of global features for HSI classification. Our model is not only optimal on OA, but also on AA and κ, which proves that our model has better stability.

**Table 5:** Classification results of 5% samples of KSC dataset.

Class	MLP	1D-CNN	M3D-DCNN	SSRN	pResNet	DBDA	SCFR	DPSCN	Proposed
1	91.39	91.14	96.74	99.33	99.63	**99.97**	98.42	99.66	**99.97**
2	82.75	85.23	82.57	98.53	95.64	98.21	90.09	96.24	**98.72**
3	84.48	88.48	77.17	**97.65**	88.13	87.04	89.65	96.00	95.52
4	50.18	58.58	46.42	90.22	72.04	86.46	58.67	86.15	**95.62**
5	45.14	54.03	45.90	**86.18**	78.06	78.33	64.17	81.18	85.42
6	44.17	46.65	64.27	96.50	86.80	95.68	79.56	87.48	**97.62**
7	78.30	71.17	76.49	91.49	92.87	89.47	85.43	**93.72**	91.49
8	83.15	85.48	81.09	99.30	97.47	99.30	95.06	98.84	**99.90**
9	91.24	93.72	93.08	99.51	99.70	**100.0**	98.42	90.00	**100.0**
10	87.16	88.65	85.76	**100.0**	98.04	99.97	97.85	99.45	**100.0**
11	94.96	93.87	99.05	**99.39**	98.89	98.17	99.02	99.23	98.81
12	86.02	88.81	91.88	99.47	99.49	99.38	92.72	98.98	**99.65**
13	99.96	99.80	**100.0**	**100.0**	**100.0**	**100.0**	99.72	**100.0**	**100.0**
OA(%)	85.17 ± 0.92	86.80 ± 0.87	87.08 ± 1.46	98.29 ± 0.59	95.88 ± 0.60	97.29 ± 1.44	92.95 ± 0.80	96.36 ± 3.16	**98.64** ± 0.40
AA(%)	78.38 ± 1.31	80.43 ± 1.18	80.03 ± 2.29	96.74 ± 1.31	92.83 ± 1.24	94.77 ± 2.60	88.37 ± 1.41	94.38 ± 2.88	**97.13** ± 0.87
κ × 100	83.48 ± 1.03	85.29 ± 0.97	85.59 ± 1.63	98.10 ± 0.66	95.42 ± 0.67	96.98 ± 1.60	92.15 ± 0.89	95.94 ± 3.52	**98.49** ± 0.44

Significant values are in bold.

**Table 6:** Classification results of 1% samples of SA dataset.

Class	MLP	1D-CNN	M3D-DCNN	SSRN	pResNet	DBDA	SCFR	DPSCN	Proposed
1	96.45	94.14	98.26	99.97	98.10	**100.0**	94.01	98.92	99.96
2	98.73	98.88	99.74	99.91	99.73	**100.0**	98.29	98.95	**100.0**
3	94.98	95.90	99.47	98.97	99.27	98.92	93.99	**100.0**	99.44
4	99.53	99.03	99.17	**99.88**	99.48	99.36	78.97	99.84	99.73
5	96.27	96.79	94.76	97.98	98.28	96.00	98.62	97.76	**99.08**
6	99.78	99.64	99.54	**100.0**	99.99	**100.0**	100.0	100.0	**100.0**
7	99.42	99.46	99.16	**99.99**	99.54	99.93	99.22	99.92	99.98
8	80.08	84.45	83.31	94.02	92.75	95.73	84.77	92.70	**96.48**
9	99.37	99.11	98.86	99.89	99.54	**100.0**	99.83	80.00	**100.0**
10	86.26	88.04	90.38	96.77	96.07	96.75	86.86	96.41	**97.36**
11	90.61	92.93	95.69	99.21	96.91	99.53	81.00	99.00	**99.80**
12	98.83	99.25	99.73	**99.98**	99.47	99.94	99.22	80.00	99.94
13	97.06	96.35	98.29	99.43	99.71	99.05	99.45	99.65	**99.89**
14	91.80	92.82	95.59	97.73	99.49	**99.67**	98.35	89.48	99.60
15	57.42	58.74	67.79	90.51	93.16	91.80	80.63	92.64	**96.35**
16	92.15	91.73	88.19	97.81	96.48	99.14	93.25	98.13	**99.31**
OA(%)	87.88 ± 0.56	89.06 ± 0.57	90.41 ± 1.35	96.98 ± 0.42	96.82 ± 0.51	97.49 ± 0.78	91.52 ± 1.82	93.74 ± 5.24	**98.50** ± 0.41
AA(%)	92.42 ± 0.41	92.95 ± 0.58	94.25 ± 0.83	98.25 ± 0.33	98.00 ± 0.42	98.49 ± 0.48	92.90 ± 3.13	95.21 ± 4.85	**99.18** ± 0.21
κ × 100	86.48 ± 0.63	87.80 ± 0.64	89.31 ± 1.51	96.64 ± 0.47	96.46 ± 0.57	97.21 ± 0.87	90.56 ± 2.04	93.06 ± 5.78	**98.33** ± 0.45

Significant values are in bold.

**Table 7:** Classification results of 1% samples of PU dataset.

Class	MLP	1D-CNN	M3D-DCNN	SSRN	pResNet	DBDA	SCFR	DPSCN	Proposed
1	85.56	92.00	94.43	98.68	93.38	98.81	95.72	98.97	**99.17**
2	95.31	96.23	97.33	99.66	99.32	99.84	98.57	**99.85**	99.82
3	60.24	75.85	70.20	87.37	65.62	89.81	84.64	96.07	**93.91**
4	81.44	89.49	93.53	97.27	95.59	96.09	93.75	**97.46**	95.53
5	99.59	99.83	98.10	**99.99**	98.86	99.80	89.80	99.98	99.79
6	75.57	86.87	83.31	99.01	95.79	**99.76**	94.26	98.70	99.74
7	73.45	77.67	78.91	97.78	80.04	**99.03**	88.68	96.26	97.71
8	77.46	79.55	87.73	95.50	88.76	97.12	94.95	97.37	**97.41**
9	99.57	99.79	96.65	**99.89**	98.73	97.74	99.47	89.77	96.31
OA(%)	86.78 ± 1.07	91.17 ± 0.47	92.24 ± 0.98	98.26 ± 0.23	94.53 ± 0.74	98.60 ± 0.33	95.72 ± 0.89	98.68 ± 0.63	**98.76** ± 0.29
AA(%)	83.13 ± 1.59	88.59 ± 0.71	88.91 ± 1.37	97.24 ± 0.32	90.68 ± 1.26	97.56 ± 0.67	93.32 ± 2.87	97.97 ± 3.19	**97.71** ± 0.62
κ × 100	82.33 ± 1.45	88.27 ± 0.61	89.66 ± 1.33	97.69 ± 0.30	92.73 ± 0.98	98.15 ± 0.44	94.31 ± 1.19	98.240.84 ±	**98.36** ± 0.39

Significant values are in bold.

**Table 8:** Classification results of 1% samples of HU dataset.

Class	MLP	1D-CNN	M3D-DCNN	SSRN	pResNet	DBDA	SC-FR	DPSCN	Proposed
1	92.74	93.74	90.40	**95.98**	91.20	93.19	90.35	94.52	93.19
2	87.24	88.15	80.04	**95.33**	92.39	92.98	89.24	90.91	95.98
3	97.28	98.13	85.07	**99.78**	91.67	99.30	83.57	99.68	98.77
4	91.79	89.65	85.91	95.18	92.36	95.41	91.55	95.65	**96.20**
5	96.42	94.85	91.50	99.35	94.76	99.65	98.81	99.51	**98.72**
6	84.65	83.52	41.35	77.61	51.01	84.40	80.57	**84.40**	82.04
7	73.41	75.91	64.54	88.05	75.97	**89.04**	76.90	84.75	88.91
8	61.81	52.99	57.38	68.70	65.31	71.20	63.15	68.56	**72.03**
9	62.75	66.38	61.66	81.94	74.79	**86.43**	74.43	81.64	84.28
10	56.02	54.58	54.46	87.05	77.59	90.82	75.57	90.26	**92.06**
11	61.79	65.74	59.06	82.22	72.33	**82.92**	68.61	81.35	83.47
12	56.38	53.42	54.72	78.49	76.99	79.41	72.01	80.50	**85.69**
13	12.16	13.16	35.38	79.54	71.70	80.54	78.76	84.47	**88.50**
14	87.37	80.31	52.89	98.97	86.80	**100.0**	98.47	89.86	98.88
15	97.99	97.65	90.70	99.91	86.25	**99.97**	99.74	99.30	99.47
OA(%)	74.89 ± 1.96	74.32 ± 1.40	69.43 ± 5.50	88.27 ± 1.61	81.27 ± 2.28	89.18 ± 2.55	81.60 ± 2.56	87.88 ± 1.57	**90.08** ± 1.43
AA(%)	74.65 ± 1.76	73.88 ± 1.37	67.00 ± 6.38	88.54 ± 2.03	80.08 ± 2.70	89.68 ± 2.01	82.78 ± 2.65	88.36 ± 2.06	**90.55** ± 1.14
κ × 100	72.83 ± 2.12	72.21 ± 1.52	66.89 ± 5.97	87.32 ± 1.75	79.74 ± 2.47	88.30 ± 2.75	80.10 ± 2.77	86.90 ± 1.70	**89.27** ± 1.54

Significant values are in bold.

**Figure 12:**
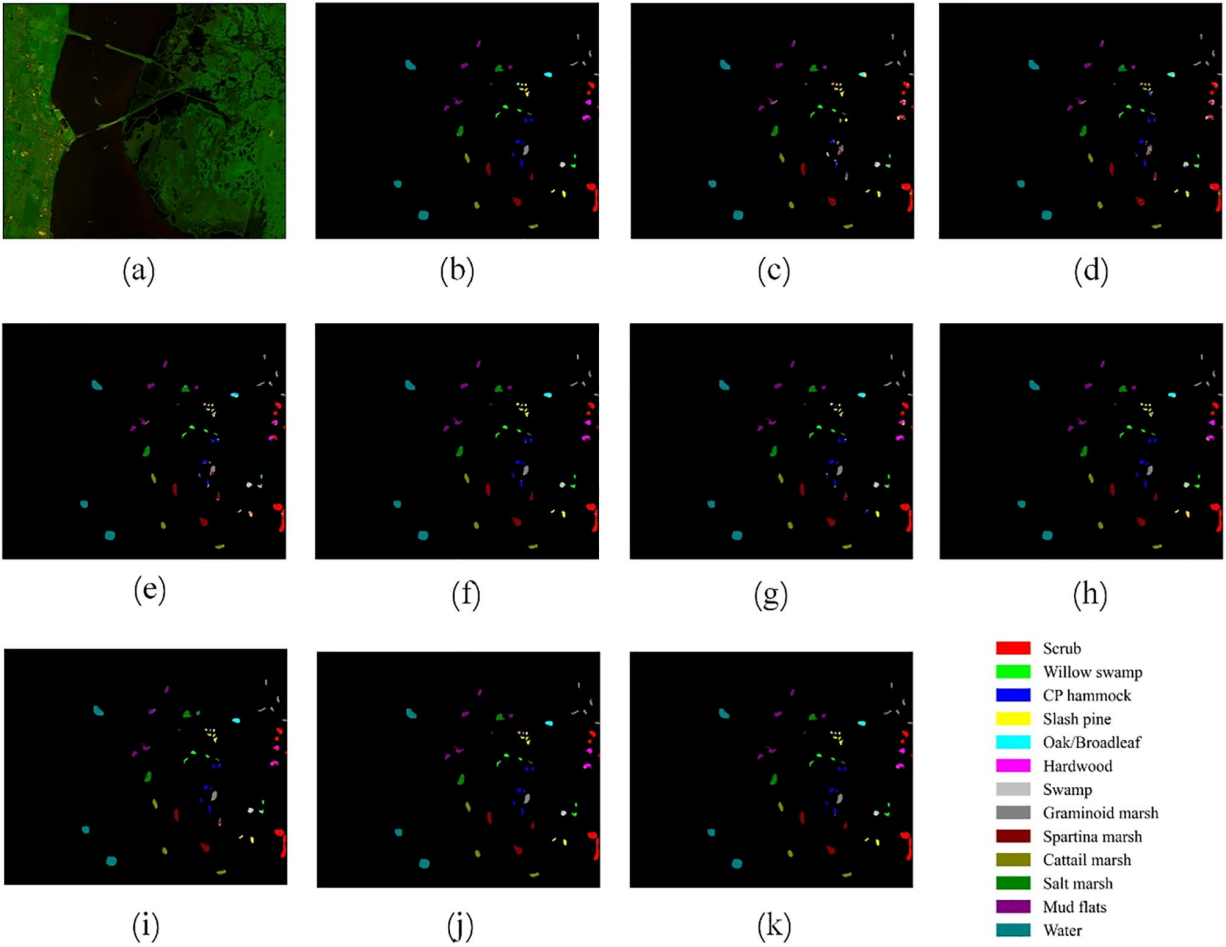
Classification maps of different models on the KSC dataset. (**a**) False-color image (**b**) Ground-truth map. (**c**) MLP. (**d**) 1D-CNN. (**e**) M3D-DCNN. (**f**) SSRN. (**g**) pResNet. (**h**) DBDA. (**i**) SCFR. (**j**) DPSCN. (**k**) DBFFT.

**Figure 13:**
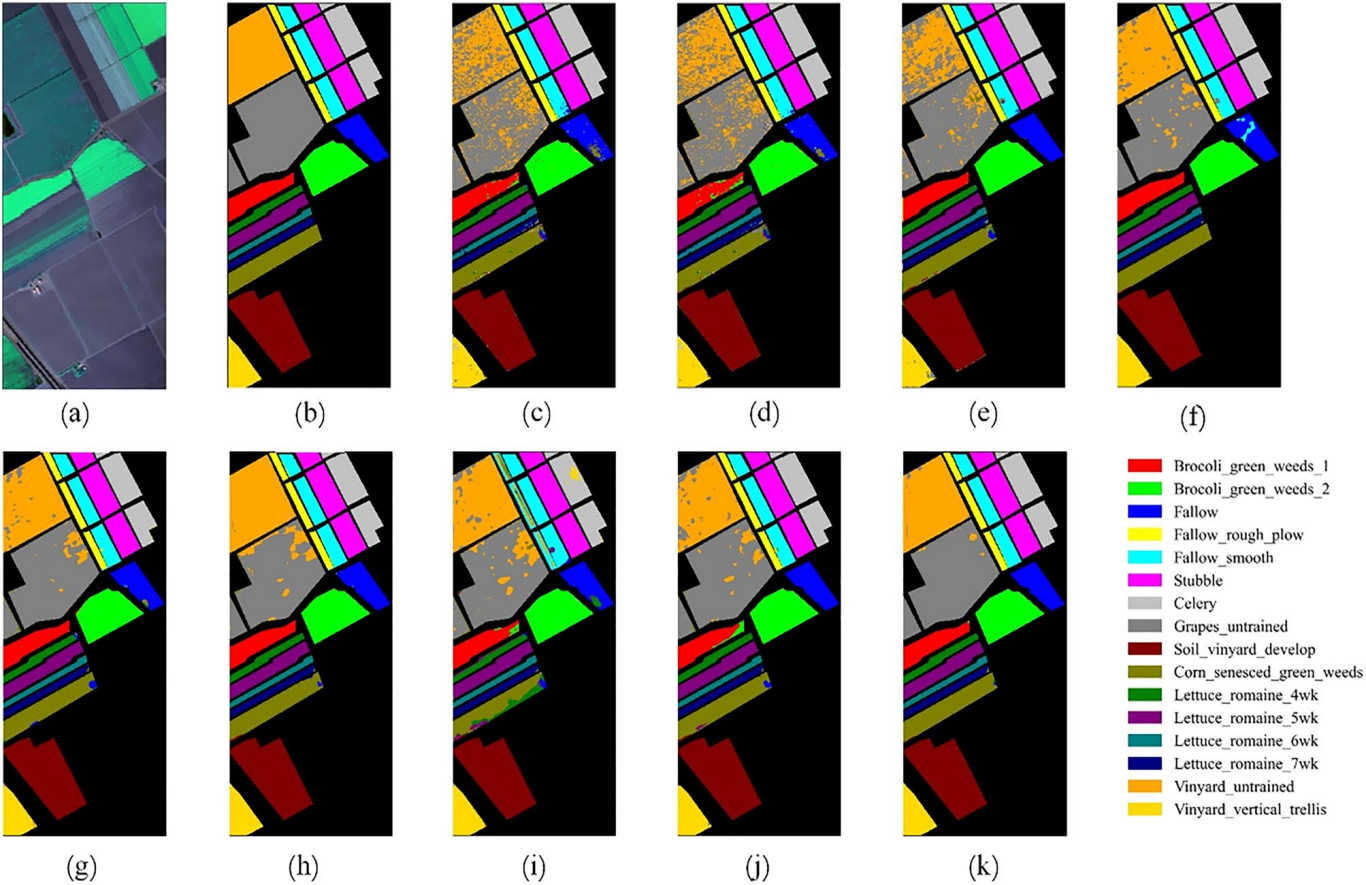
Classification maps of different models on the SA dataset. (**a**) False-color image. (**b**) Ground-truth map. (**c**) MLP. (**d**) 1D-CNN. (**e**) M3D-DCNN. (**f**) SSRN. (**g**) pResNet. (**h**) DBDA. (**i**) SCFR. (**j**) DPSCN. (**k**) DBFFT.

**Figure 14:**
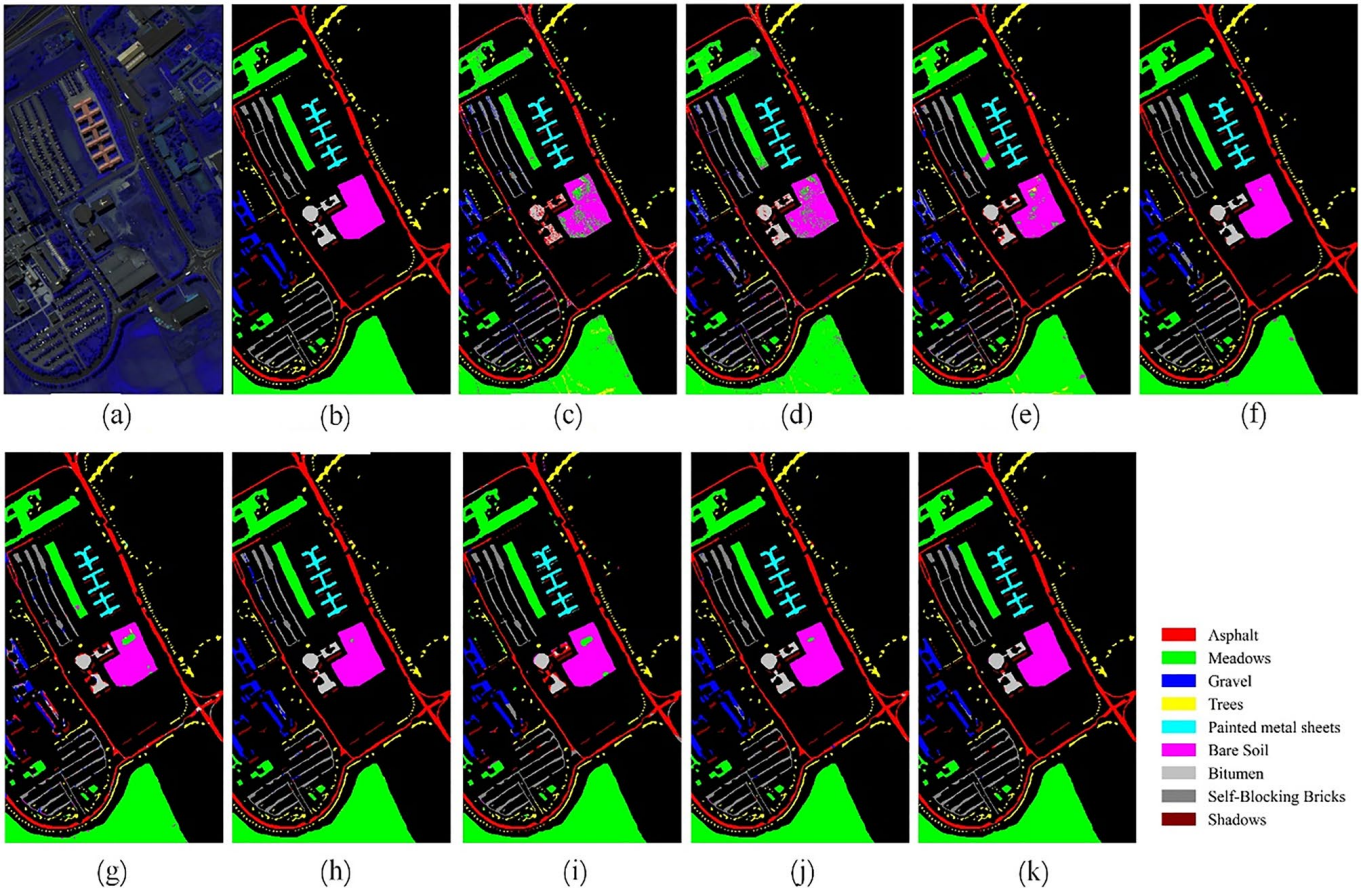
Classification maps of different models on the PU dataset. (**a**) False-color image. (**b**) Ground-truth map. (**c**) MLP. (**d**) 1D-CNN. (**e**) M3D-DCNN. (**f**) SSRN. (**g**) pResNet. (**h**) DBDA. (**i**) SCFR. (**j**) DPSCN. (**k**) DBFFT.

**Figure 15:**
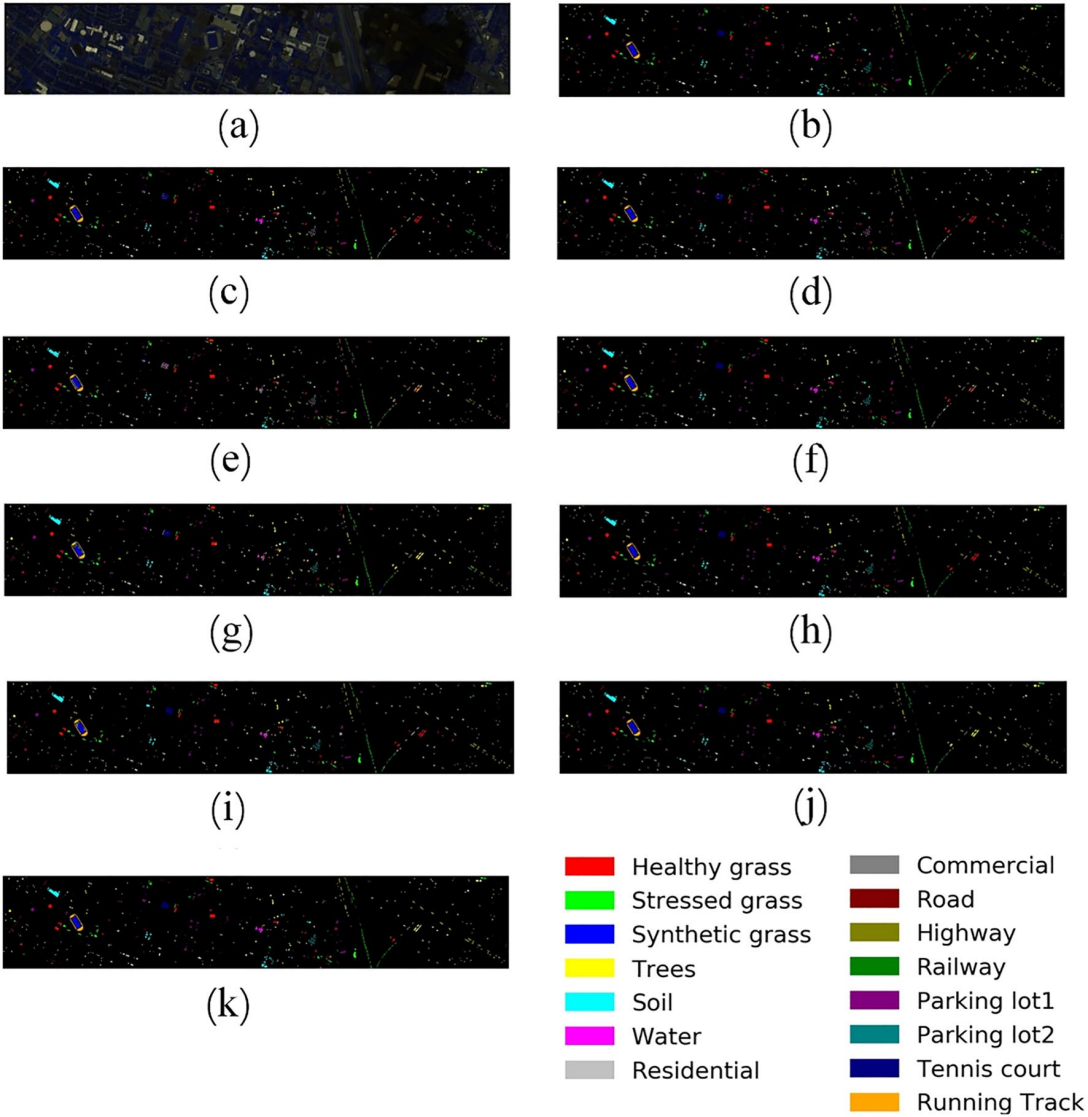
Classification maps of different models on the HU dataset. (**a**) False-color image. (**b**) Ground-truth map. (**c**) MLP. (**d**) 1D-CNN. (**e**) M3D-DCNN. (**f**) SSRN. (**g**) pResNet. (**h**) DBDA. (**i**) SCFR. (**j**) DPSCN. (**k**) DBFFT.

Figures 12, 13, 14 and 15 show the original false-color image of the HSI, the ground truth map, the classification maps of DBFFT, and all the compared methods. We can find that there is a lot of salt and pepper noise on the classification maps of MLP and 1DCNN that only use spectral information for classification. The classification map of the CNN-Based model based on spectral and spatial information and the classification map of our proposed model are more smooth. However, M3D-DCNN has worse classification results than pResNet, SSRN, DBDA, SCFR, DPSCN, and DBFFT due to its severe overfitting. Our proposed model extracts global spectral features and global spatial features by introducing a self-attention mechanism, and fuses spectral and spatial features through a feature fusion layer to obtain a very smooth and ideal classification map. Compared with all other models, our classification map generates the least noise on the four datasets, and the classification map is the most accurate and smooth.

**Figure 16:**
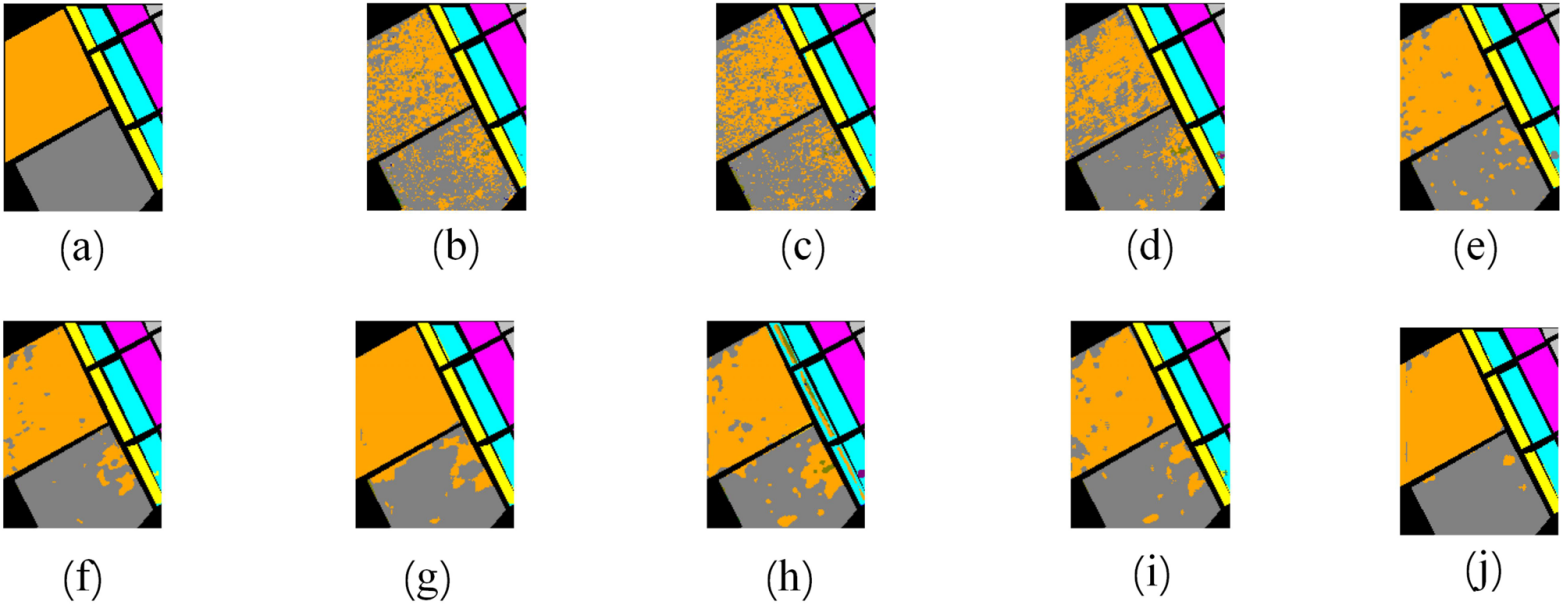
Part of the classification map for different models on the SA dataset. (**a**) Ground-truth map. (**b**) MLP. (**c**)1D-CNN. (**d**) M3D-DCNN. (**e**) SSRN. (**f**) pResNet. (**g**) DBDA. (**h**) SCFR. (**i**) DPSCN. (**j**) DBFFT.

Figure 16 shows a part of the SA classification map, and we can see that in the case of small training set samples, class 8 and class 15 are extremely prone to misclassification on both our proposed model and the comparison model. MLP, 1D-CNN and M3D-DCNN misclassify a lot of these two classes. Our proposed model has the least number of misclassifications on class 8 and class 15 compared to other models, which is the performance of our proposed model in the face of overfitting.

Table 9 reports the training time and test time of the proposed model and 5 models with similar performance. It can be seen that our model outperforms DBDA and SSRN in both training time. On the SA dataset, the training time of SSRN is 3 times that of ours, and the training time of DBDA is 2 times that of us. Compared with DPSCN and SCFR, our model requires more training time and testing time, but DPSCN and SCFR can only achieve similar performance to our proposed model on some datasets, and perform poorly on other datasets. For example on the SA dataset, the OA of DPSCN and SC-FR is 4.76% and 6.98% lower than our proposed model, respectively. We thought it was worth the extra time to get better performance.

**Table 9:** Training time, and test time for different models on the four data sets.

	SSRN	pResNet	DBDA	SC-FR	DPSCN	Proposed
**PU**						
Training time (s)	194.83	61.52	170.67	8.60	37.74	117.35
Test time (s)	10.41	7.84	21.78	3.14	4.65	13.09
**KSC**						
Training time (s)	218.54	38.85	199.82	5.81	26.11	187.97
Test time (s)	1.59	0.93	3.46	0.41	0.52	3.23
**SA**						
Training time (s)	566.60	82.41	342.82	10.92	50.51	165.67
Test time (s)	19.73	12.68	44.84	5.10	7.18	21.61
**HU**						
Training time (s)	98.39	24.60	103.03	3.79	15.53	62.09
Test time (s)	4.37	2.70	9.16	0.99	1.57	5.71

### Investigation of training sample

**Figure 17:**
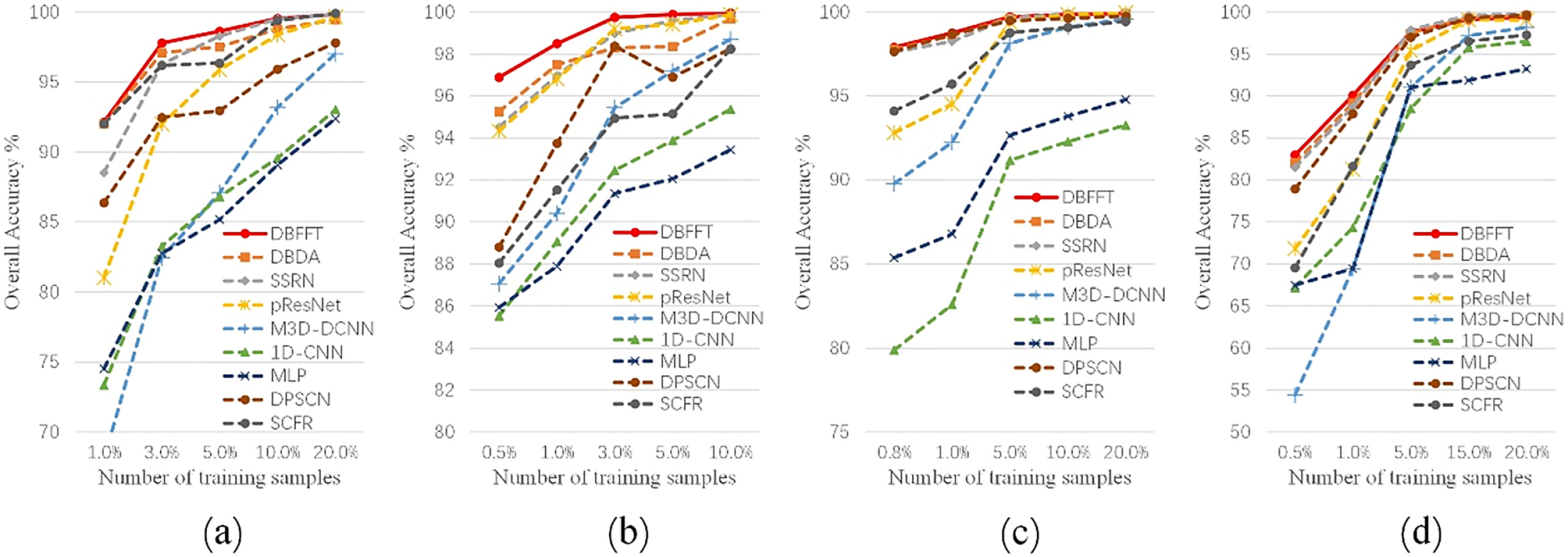
OA (%) of DBFFT with different number of training samples in the four datasets. (**a**) KSC dataset. (**b**) SA dataset. (**c**) PU dataset. (**d**) HU dataset.

The excellent performance of deep learning methods relies on a large number of labeled datasets, but it is usually difficult to obtain enough labeled data for HSI. Therefore, we test the performance of our proposed model and all compared models under different numbers of training set samples. For KSC, we take 1%, 3%, 5%, 10%, and 20% of labeled pixels as training samples. For PU, we choose 0.8%, 1%, 5%, 10%, and 20% of labeled pixels as training samples. For SA, we consider 0.5%, 1%, 3%, 5%, and 10% of labeled pixels as training samples. For HU, we consider 0.5%, 1%, 5%, 15%, and 20% of labeled pixels as training samples. As shown in Fig. 17, as the training samples increase, the OA of all models also increases. In the case of large training samples, all performances of SSRN, DBDA, pResNet and our proposed model are close to perfect. But when the training samples are reduced, our proposed model consistently outperforms other models. It should be mentioned that our proposed model has the highest accuracy on all sample proportions of SA, and it only performs suboptimally at 20% sample proportion on PU and KSC datasets. Considering the difficulty of sample acquisition of HSI, our proposed model is more suitable for the actual situation.

### Effect of label smooth

**Figure 18:**
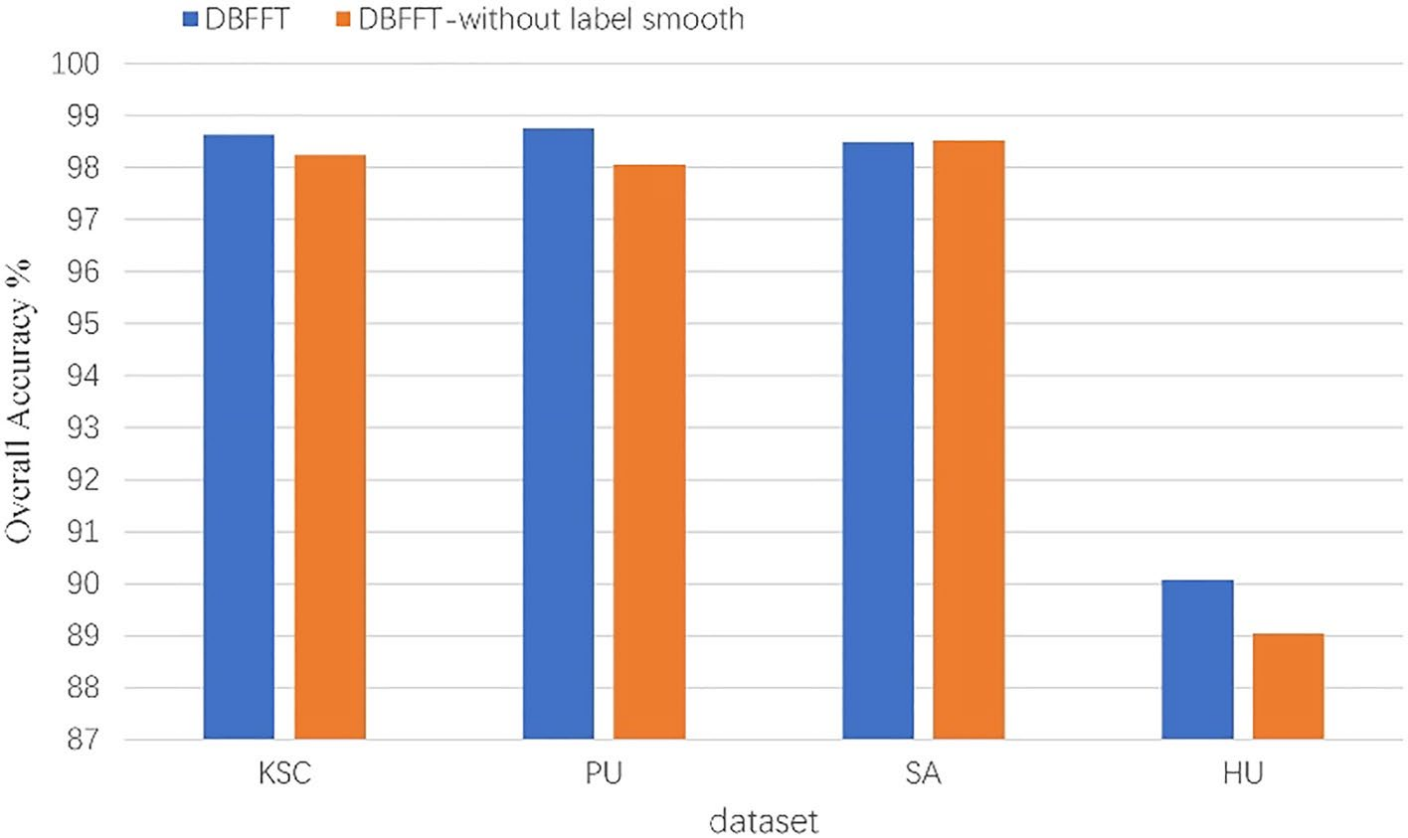
The effect of label smooth on the performance of the model.

To verify the effect of label smooth on model training, we retrain the models with label smooth removed and compare their performance. The results are shown in Fig. 18. On the four datasets, the performance of the model will be improved by adding label smooth during training. It proves that the model combined with label smooth has stronger generalization ability.

### Effect of feature fusion layer

**Figure 19:**
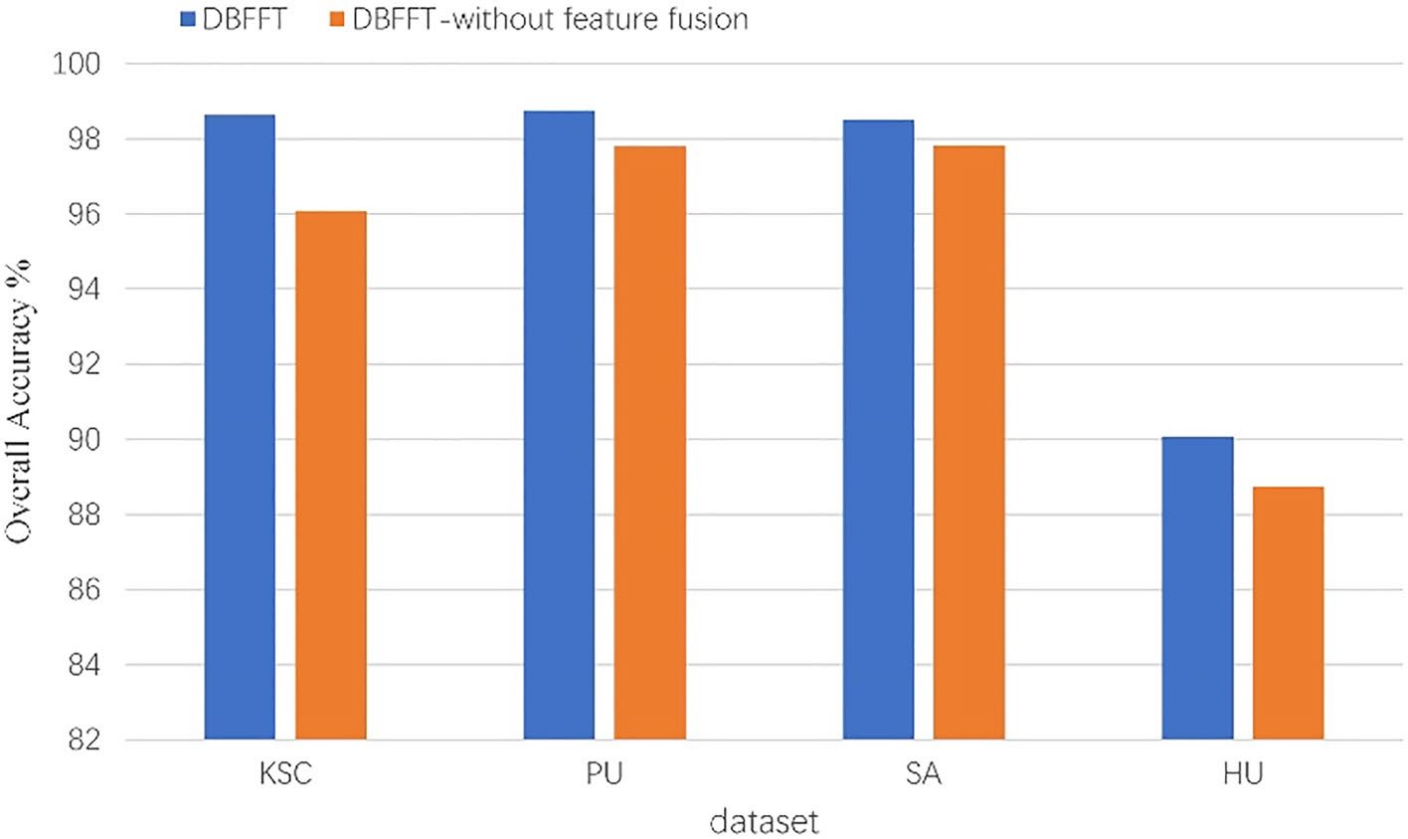
The effect of feature fusion on the performance of the model.

In this section, we will compare the performance of the proposed model with that model not having feature fusion layer. The results are shown in Fig. 19. We can see that feature fusion significantly improves the performance of the model on all four datasets, which proves that feature fusion layer improves the performance of the model by fusing the spectral and spatial features of HSI.

### Effect of attention mechanism

**Figure 20:**
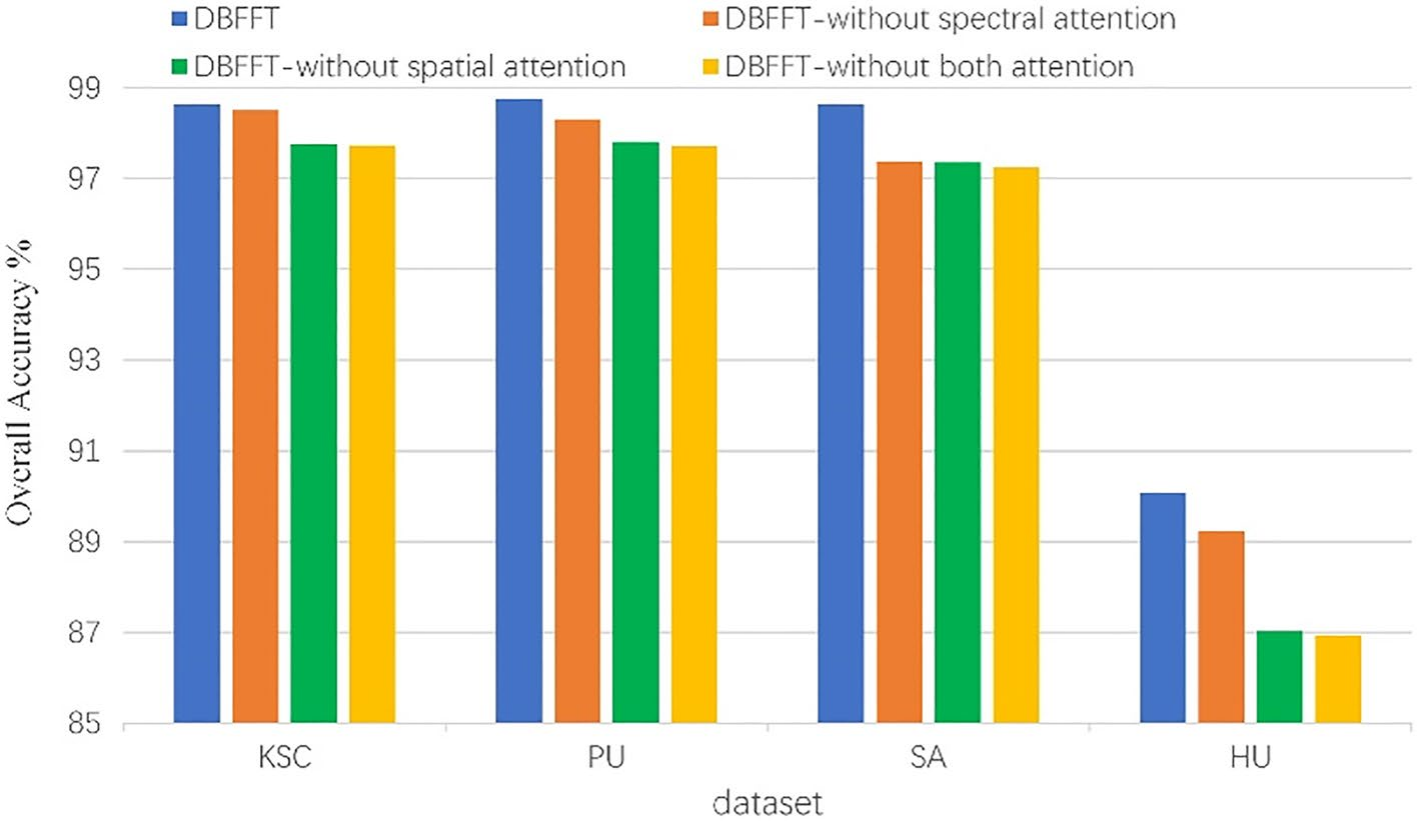
The effect of attention mechanism on the performance of the model.

We verify the effectiveness of the attention mechanism by removing the spectral attention module, spatial attention module, and removing both attention modules from the model respectively. The experimental results are shown in Fig. 20. We can see that the performance of the model on all four datasets decreases significantly when both modules are removed, and the performance of the model is reduced by 0.91%, 1.04%, 1.26%, and 3.61% on KSC, PU, SA, and HU, respectively. After only removing the spatial attention module, the performance of the model is reduced by 0.88%, 0.95%, 1.2%, and 3.44% on KSC, PU, SA, and HU, respectively. It is revealing that the spatial attention module plays a major role in improving the performance of the model. When we remove the spectral attention module, the results show that it has a certain but non-significant impact on the performance of the model. Therefore, we can conclude that the model can improve the performance of the model after adding the attention mechanism.

## Conclusion

In this paper, we propose a Double-Branch feature fusion Transformer (DBFFT) model for HSI classification. The proposed model can overcome the shortcomings of CNN-based models, which are not good at learning the long-distance dependency relations of spectral bands and extracting global spatial features of HSI. We firstly present two attention mechanism modules to extract spectral and spatial features separately. According to the characteristics of HSI, we adopt Pixel-wise embedding and Band-wise embedding on the spectral branch and spatial branch to process the feature maps to better utilize the self-attention mechanism to extract the global spatial and global spectral features of HSI. Then, we design a feature fusion layer to fuse the spectral and spatial features of the two branches. In view of the limited number of training samples of HSI, our model can outperform the CNN-based model in the case of small samples. In addition, we also employ the label smooth technique to improve the generalization ability of the model in small sample scenarios.

In the future, we will do more works to improve the proposed model to achieve more effectiveness and performance. The first work is to improve the structure of the proposed model to enhance its ability to extract global features and generalization. Another is to improve the fusion ability of the spectral and spatial features with a more effective feature fusion layer. Finally, more hyperspectral image datasets could be considered, not just these few public datasets.

## Data Availability

The data that support the findings of this study are available from the Grupo de Inteligencia Computacional (GIC) website (http://www.ehu.eus/ccwintco/index.php/Hyperspectral_Remote_Sensing_Scenes).

## References

[1] Landgrebe D (2002). Hyperspectral image data analysis. IEEE Signal Process. Mag..

[2] Fauvel M, Tarabalka Y, Benediktsson JA, Chanussot J, Tilton JC (2013). Advances in spectral-spatial classification of hyperspectral images. Proc. IEEE.

[3] Li J, Marpu PR, Plaza A, Bioucas-Dias JM, Benediktsson JA (2013). Generalized composite kernel framework for hyperspectral image classification. IEEE Trans. Geosci. Remote Sens..

[4] Ibrahim A (2018). Atmospheric correction for hyperspectral ocean color retrieval with application to the Hyperspectral Imager for the Coastal Ocean (HICO). Remote Sens. Environ..

[5] Mahesh S, Jayas D, Paliwal J, White N (2015). Hyperspectral imaging to classify and monitor quality of agricultural materials. J. Stored Prod. Res..

[6] Haboudane D, Miller JR, Pattey E, Zarco-Tejada PJ, Strachan IB (2004). Hyperspectral vegetation indices and novel algorithms for predicting green LAI of crop canopies: Modeling and validation in the context of precision agriculture. Remote Sens. Environ..

[7] Manjunath K, Ray S, Vyas D (2016). Identification of indices for accurate estimation of anthocyanin and carotenoids in different species of flowers using hyperspectral data. Remote Sens. Lett..

[8] Han Y, Li J, Zhang Y, Hong Z, Wang J (2017). Sea ice detection based on an improved similarity measurement method using hyperspectral data. Sensors.

[9] Paoletti ME, Haut JM, Plaza J, Plaza A (2019). Deep learning classifiers for hyperspectral imaging: A review. ISPRS J. Photogramm. Remote Sens..

[10] Fauvel M, Benediktsson JA, Chanussot J, Sveinsson JR (2008). Spectral and spatial classification of hyperspectral data using SVMs and morphological profiles. IEEE Trans. Geosci. Remote Sens..

[11] Hongwei Z, Basir O (2005). An adaptive fuzzy evidential nearest neighbor formulation for classifying remote sensing images. IEEE Trans. Geosci. Remote Sens..

[12] Collobert R, Bengio S (2004). Links between perceptrons, MLPs and SVMs. Proc. ICML.

[13] Benediktsson JA, Palmason JA, Sveinsson JR (2005). Classification of hyperspectral data from urban areas based on extended morphological profiles,". IEEE Trans. Geosci. Remote Sens..

[14] Li W, Du Q (2014). Gabor-filtering-based nearest regularized subspace for hyperspectral image classification. IEEE J. Select Topics Appl. Earth Observ. Remote Sens..

[15] Okan, A., Özdemir, B., Gedik, B.E., Yasemin, C. & Çetin, Y. Hyperspectral classification using stacked autoencoders with deep learning. In *Proc.WHISPERS.* 1–4 (2014).

[16] Zhou F, Hang R, Liu Q, Yuan X (2019). HSI classification using spectral-spatial LSTMs. Neurocomputing.

[17] Hang R, Liu Q, Hong D, Ghamisi P (2019). Cascaded recurrent neural networks for hyperspectral image classification. IEEE Trans. Geosci. Remote Sens..

[18] Larochelle, H. & Bengio, Y. Classification using discriminative restricted boltzmann machines. In *Proc. ICML*. 536–543 (2008).

[19] Hong D (2022). SpectralFormer: Rethinking hyperspectral image classification with transformers. IEEE Trans. Geosci. Remote Sens..

[20] Wei Hu, Huang Y, Wei Li, Zhang F, Li H (2015). Deep convolutional neural networks for hyperspectral image classification. J. Sens..

[21] Zhong Z, Li J, Luo Z, Chapman M (2018). Spectral-spatial residual network for hyperspectral image classification: A 3-D deep learning framework. IEEE Trans. Geosci. Remote Sens..

[22] Paoletti ME, Haut JM, Fernandez-Beltran R, Plaza J, Plaza AJ, Pla F (2019). Deep pyramidal residual networks for spectral-spatial hyperspectral image classification. IEEE Trans. Geosci. Remote Sens..

[23] Dongyoon, H., Kim, J., & Kim, J. Deep pyramidal residual networks. In *Proc. CVPR.* 5927–5935 (2017).

[24] Rui L, Zheng S, Duan C, Yang Y, Wang X (2020). Classification of hyperspectral image based on double-branch dual-attention mechanism network. Remote Sens..

[25] Gao H, Yang Y, Li C, Zhang X, Zhao J, Yao D (2019). Convolutional neural network for spectral-spatial classification of hyperspectral images. Neural Comput..

[26] Dang L, Pang P, Zuo X, Liu Y, Lee J (2021). A dual-path small convolution network for hyperspectral image classification. Remote Sens..

[27] Chang Y-L (2022). Consolidated convolutional neural network for hyperspectral image classification. Remote Sens..

[28] Shi H, Cao G, Zhang Y, Ge Z, Liu Y, Fu P (2022). *H*^*2*^*A*^*2*^Net: A hybrid convolution and hybrid resolution network with double attention for hyperspectral image classification. Remote Sensing..

[29] He X, Chen Y, Lin Z (2021). Spatial-spectral transformer for hyperspectral image classification. Remote Sens..

[30] Vaswani, A. et al. Attention is all you need. arXiv preprint arXiv:1706.03762 (2017).

[31] He J, Zhao L, Yang H, Zhang M, Li W (2020). HSI-BERT: Hyperspectral image classification using the bidirectional encoder representation from transformers. IEEE Trans. Geosci. Remote Sens..

[32] Dosovitskiy, A. et al. An image is worth 16×16 words: Transformers for image recognition at scale. arXiv preprint arXiv:2010.11929 (2020).

[33] Yuan, K., Guo, S., Liu, Z., Zhou, A., Yu F., & Wu, W. Incorporating convolution designs into visual transformers. In *Proc. ICCV*. 579–588 (2021).

[34] Chen C. F. R., Fan, Q. & Panda, R. Crossvit: Cross-attention multi-scale vision transformer for image classification. In *Proc. ICCV.* 357–366 (2021).

[35] Hu J., Shen, L. & Sun, G. Squeeze-and-excitation networks. In *Proc. CVPR.* 7132–7141. (2018).

[36] Zhu M, Jiao L, Liu F, Yang S, Wang J (2021). Residual spectral-spatial attention network for hyperspectral image classification. IEEE Trans. Geosci. Remote Sens..

[37] Sanghyun, W., Park, J., & Lee, J.-Y. CBAM: Convolutional block attention module. In *Proc. ECCV.* 3–19 (2018).

[38] Kayhan O. S. & Gemert, J. C. V. On translation invariance in CNNs: Convolutional layers can exploit absolute spatial location. In *Proc. CVPR*. 14274–14285 (2020).

[39] Acito N, Matteoli S, Rossi A, Diani M, Corsini G (2016). Hyperspectral airborne “Viareggio 2013 Trial” data collection for detection algorithm assessment. IEEE J. Select. Topics Appl. Earth Observ. Remote Sens..

[40] Donoho DL (2000). High-dimensional data analysis: The curses and blessings of dimensionality. AMS Math Chall. Lect..

[41] He M, Li B, Chen H (2017). Multi-scale 3D deep convolutional neural network for hyperspectral image classification. Proc. ICIP.

